# Diverse phage communities are maintained stably on a clonal bacterial host

**DOI:** 10.1126/science.adk1183

**Published:** 2024-12-12

**Authors:** Nora C. Pyenson, Asher Leeks, Odera Nweke, Joshua E. Goldford, Jonas Schluter, Paul E. Turner, Kevin R. Foster, Alvaro Sanchez

**Affiliations:** 1 Institute for Systems Genetics, New York University Grossman School of Medicine; New York, USA; 2 Department of Microbiology, New York University Grossman School of Medicine; New York, USA; 3 Department of Ecology and Evolutionary Biology, Yale University; New Haven, USA; 4 Quantitative Biology Institute, Yale University; New Haven, USA; 5 Division of Geological and Planetary Sciences, California Institute of Technology; Pasadena, USA; 6 Laura and Isaac Perlmutter Cancer Center, New York University Grossman School of Medicine, New York, NY, USA; 7 Program in Microbiology, Yale School of Medicine; New Haven, USA; 8 Center for Phage Biology & Therapy, Yale University; New Haven, USA; 9 Department of Biology, University of Oxford; Oxford, UK; 10 Department of Biochemistry, University of Oxford; Oxford, UK; 11 Sir William Dunn School of Pathology, University of Oxford; Oxford, UK; 12 Institute of Functional Biology & Genomics, CSIC & University of Salamanca, Salamanca, Spain

## Abstract

Bacteriophages are the most abundant and diverse biological entities on Earth, yet the ecological mechanisms that sustain this extraordinary diversity remain unclear. Here, we have discovered that phage diversity generically outstrips the diversity of their bacterial hosts under simple experimental conditions. We assembled and passaged dozens of diverse phage communities on a single, non-evolving strain of *Escherichia coli* until the phage communities reached equilibrium. In all cases, we found that two or more phage species coexisted stably, despite competition for a single, clonal host population. Phage coexistence was supported through host phenotypic heterogeneity, whereby bacterial cells adopting different growth phenotypes served as niches for different phage species. Our experiments reveal that a rich community ecology of bacteriophages can emerge on a single bacterial host.

The diversity of bacteriophages is staggering. An individual human gut contains hundreds of different phage species that stably persist over years (1). It is now routine to discover thousands of new phage species across virtually every type of microbial community, from the human-associated microbiome to the hot springs at Yellowstone National Park (2, 3). Phage abundances can be similarly vast. A single gram of soil contains over one billion phage virions (4), with an estimated 10^31^ total virions on Earth (5). This abundance and diversity underpins the essential role of phage in microbial community dynamics, marine ecosystem nutrient cycling, and even human health due to their effects on our microbiomes (6).

A key explanation for phage diversity is based upon the diversity of their hosts. Each phage species infects a narrow bacterial host range, so microbial communities with more bacterial diversity support more phage diversity (7). Moreover, experiments show that communities with multiple bacterial species (8), or even multiple bacterial strains of a single species (9, 10), can support the coexistence of diverse phage species. These findings have led to the widespread assumption that phage diversity is constrained by the genetic diversity of the resident host population (11, 12). Mechanisms such as host diversification or spatial isolation (13) would be necessary for multiple phage species to coexist, otherwise competitive exclusion would drive the domination of a single phage species (14, 15). If correct, the number of bacterial genotypes would set an upper limit on phage diversity within a given community.

Here we experimentally tested whether host genetic diversity is indeed required for the maintenance of diverse phage species. We generated diverse populations of naturally isolated phage species and tested for stable coexistence on a clonal, non-evolving, bacterial host (*E. coli*) under standard laboratory conditions. By removing host genetic diversity and spatial structure, we eliminated the typically-discussed (12) mechanisms that can support phage diversity. In doing so, we established a conservative, lower bound on the amount of phage diversity that can persist in the face of competition for a single host.

## Results

### Lytic phages assembled into diverse communities

**Fig. 1:**
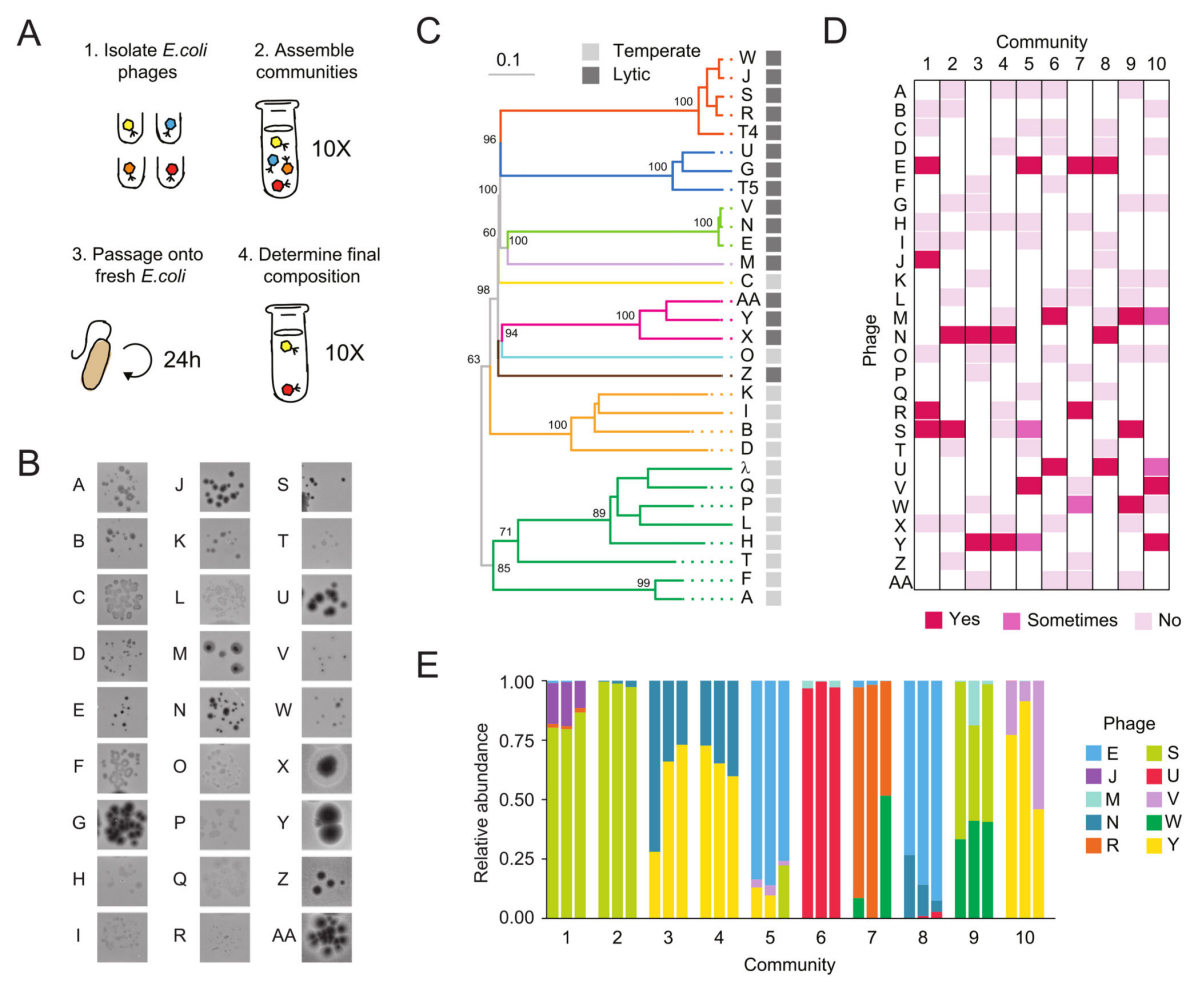
Phage communities were diverse, common, and predictable on a single host. (**A**) Illustration of the experimental workflow. (1) Isolate and genome sequence 27 *E. coli* phage species from natural samples. (2) Infect *E. coli* with 10 different starting communities each containing a random set of 10 species from our collection. (3) Filter, dilute, and passage the phage community onto fresh bacteria every 24 hours. (4) At day 12 determine the composition through deep sequencing and plaque morphologies. (**B**) Diverse plaque morphologies of the species in our collection, with each species given a different alphabetical name. (**C**) Phylogenetic tree including all 27 phage species in our collection and model phages T4, T5 and lambda as references. The numbers above the branches are GBDP pseudo-bootstrap support values from 100 replications and the branch length is scaled by the D0 formula. The branches were colored based on family classification (see fig. S1 or table 1 for family names). (**D**) Every community contained multiple diverse phage species at the final passage, as determined through deep sequencing (N = 3 biological replicates). “No” indicated that the species was present in the starting community but was not detectable at the final passage in any biological replicate. “Sometimes” indicated that the species was present in some replicates, while “Yes” indicated that the species was present in all replicates. (**E**) Communities differed in their composition and relative abundance of species, but were similar across the three biological replicates for the same community.

To test whether competitive exclusion generically limits phage diversity to a single phage species per host strain, we first isolated a diverse collection of naturally occurring phage species capable of infecting the *E. coli* K-12 strain BW25113 (Fig. 1A). These *E. coli* phage species were isolated from different environmental samples, including animal droppings and river water (collected in New Haven, Connecticut, USA), that generated clearings on a lawn of *E. coli*. Individual phage species were then plaque purified and their genomes were sequenced. This yielded a collection of 27 double-stranded DNA tailed bacteriophage species (Caudoviricetes) (16) that was diverse phylogenetically, phenotypically, and in terms of replication strategies (i.e. life history). Most species had distinct plaque morphologies (Fig. 1B) and shared limited genome sequence homology with one another, apart from species in the same family (fig. S1). The genetic divergence reflected the fact that our collection was taxonomically diverse (17) (Fig. 1C and table S1), containing 10 families and 13 different genera. Fourteen species were obligately lytic, meaning that they only reproduced by killing host cells, and the remaining 13 were temperate species, meaning that they could either transmit vertically when the host cell reproduces or reproduce as a lytic phage (Fig. 1C and fig. S2). Each of the 27 species was given a unique short-hand alphabetical name (phage A to Z, and phage AA) (Fig. 1B, C, table S1).

With this diverse collection of phage species we performed top-down community assembly experiments to test for phage coexistence among 10 different phage communities on a single non-evolving host population (Fig. 1A). At passage 0, we mixed random subsets of 10 different phage species from the collection (10^5^ plaque forming units (pfu) per species) with 1.2 x 10^7^ *E. coli* (N =3 biological replicates). Every 24 hours the phage component was filtered to eliminate any surviving cells and diluted onto fresh *E. coli* cells. This ensured that the bacterial population did not evolve or acquire genetic diversity throughout the passaging regime. At the twelfth passage, we determined which phage species remained by shotgun sequencing each community, assembling phage genomes de novo, and then comparing the assembled genomes to our phage collection (see Methods). We validated that the deep sequencing accurately measured the relative abundance and identity of each species by quantifying plaque morphologies through top agar plating of three communities (RMSD = 0.17, N = 9) (fig. S3).

We found that every single passaging experiment led to phage coexistence. Despite competition for a shared host population, all phage communities contained between two and four coexisting phage species at the final passage (Figs. 1D and 1E and figs. S4 and S5A). Every community was genetically diversity, with at least two community members from different families (S5A). Our final phage communities also exhibited a significant beta diversity: eight out of 10 communities had different sets of species, while the other two communities (3 and 4) both contained the same two species (phages N and Y). The passaging regime was highly reproducible in assembling phage communities: for seven of 10 communities, all three biological replicates had assembled into the exact same phage species composition. The other three final communities (5, 7, and 10), each shared at least two species between their biological replicates, but differed in the identity or presence of a third species. The phage replication strategy was critical for this emergent diversity, since all the species remaining at the final passage were lytic (fig. S5A).

To test whether our finding of generic multi-phage coexistence was robust, we repeated the experiment with the following modifications: (i) allowing for host co-evolution or; (ii) reducing the starting phage diversity. Using six of the same starting communities from Fig. 1D, we repeated the experiment without filtering between passages to allow for spontaneously phage-resistant mutant cells to carry over. We again found phage coexistence in all communities (fig. S5B). Next, we tested communities that contained five randomly chosen phage species, instead of 10, with at least two lytic species in each starting community. After passaging with filtering between passages, we again saw that coexistence was ubiquitous (fig. S5C).

In total, from all our passaging experiments we found that 13 of the 14 lytic species in our collection were present in at least one community (fig. S5 and table S1). This indicated that virtually all the lytic species had the potential for coexistence, albeit not in every community. The absence of coexisting temperate species suggested they were excluded by other species in a community. However, it was possible that the daily passaging regime could have driven these species to extinction even in the absence of other community members. We tested this hypothesis by passaging eight temperate species in isolation. Seven of eight species persisted at high titers greater than 10^5^ pfu/ul (fig. S6A), which was above the limit of detection for most of our communities (fig. S6B). This indicated that the temperate species could persist passaging when other species were absent from the culture. Competition from lytic species likely drove their extinction since temperate species typically produce fewer virions (18).

### Stable lytic phage communities assembled rapidly

**Fig. 2:**
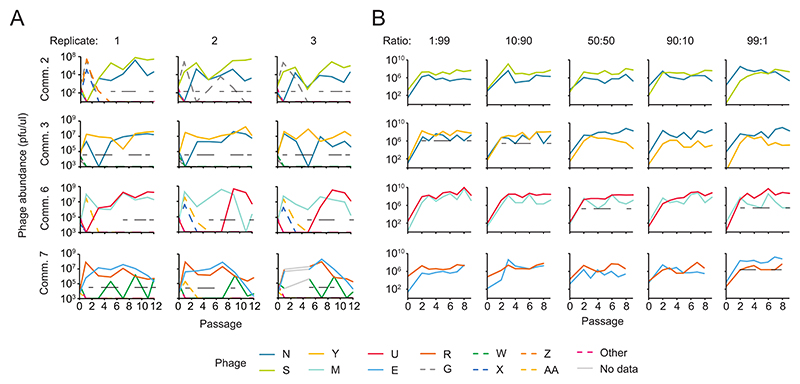
Diversity was stable throughout community passaging. Phage species abundances over time for communities 2, 3, 6, and 7 (top to bottom), quantified using plaque morphologies. (**A**) The three biological replicates (labelled above the charts) of four communities from Fig. 1D showed the early dominance of final community members. The average limit of detection for each replicate is indicated by the black dotted line or is otherwise indicated by the x-axis. Phage species shown with dashed lines were absent from the final community. Phage abundances were quantified on passage 0 and then every other passage starting at passage 1 until passage 11 and 12. (**B**) Reconstituted 2-member communities maintained both phage species irrespective of starting proportion. The initial relative abundances of each phage species varied from ∼1% to ∼99% of the total phage population and are listed above the charts. From top to bottom these represent the ratio of phage N:S, N:Y, M:U, and E:R. For simplicity we reconstituted community 7 with only phages E and R, although the original communities also had phage W. The limit of detection is only shown for replicates where one of the two members was undetectable at least once during passaging. Phage abundances were measured through plaquing on passage 0 and then every day starting at passage 2 until passage 9 (communities 2, 3, 6) or passage 8 (community 7).

To investigate how quickly our communities of lytic phage species assembled we quantified each species’ abundance during passaging for four random communities from Fig. 1 using plaque morphologies. The most abundant species at the final passage usually became (and remained) dominant starting at passage 3 (Fig. 2A). In addition, most species that were absent from the final communities were lost by passage 3. These results are consistent with rapid ecological dynamics that arise early during passaging and likely reflect differences in phage growth rates. However, in a few cases, including the replicates of community 7, the phage species constituting the final community did not become dominant until later passages, indicating other processes occurred. In all communities, the absolute population size of each species fluctuated, but the overall diversity and composition remained relatively stable.

The speed at which community composition became fixed suggested that our phage communities would be stable to perturbations. We directly evaluated community stability by testing whether each species could invade the community when initially present as a rare proportion of the population. We reconstituted and passaged the four different 2-member communities examined above (Fig. 2A) and varied the starting proportion of each species between 1%, and 99% of the total population (Fig. 2B). We found that both species were detectable at the last passage in every condition for all four communities, apart from one of the conditions for community 6. Each species in these communities increased in frequency when rare and decreased in frequency when abundant (fig. S7). This negative frequency dependent selection maintained stable coexistence after perturbations in each species relative abundance (19).

### Phage-phage interactions were largely antagonistic

**Fig. 3:**
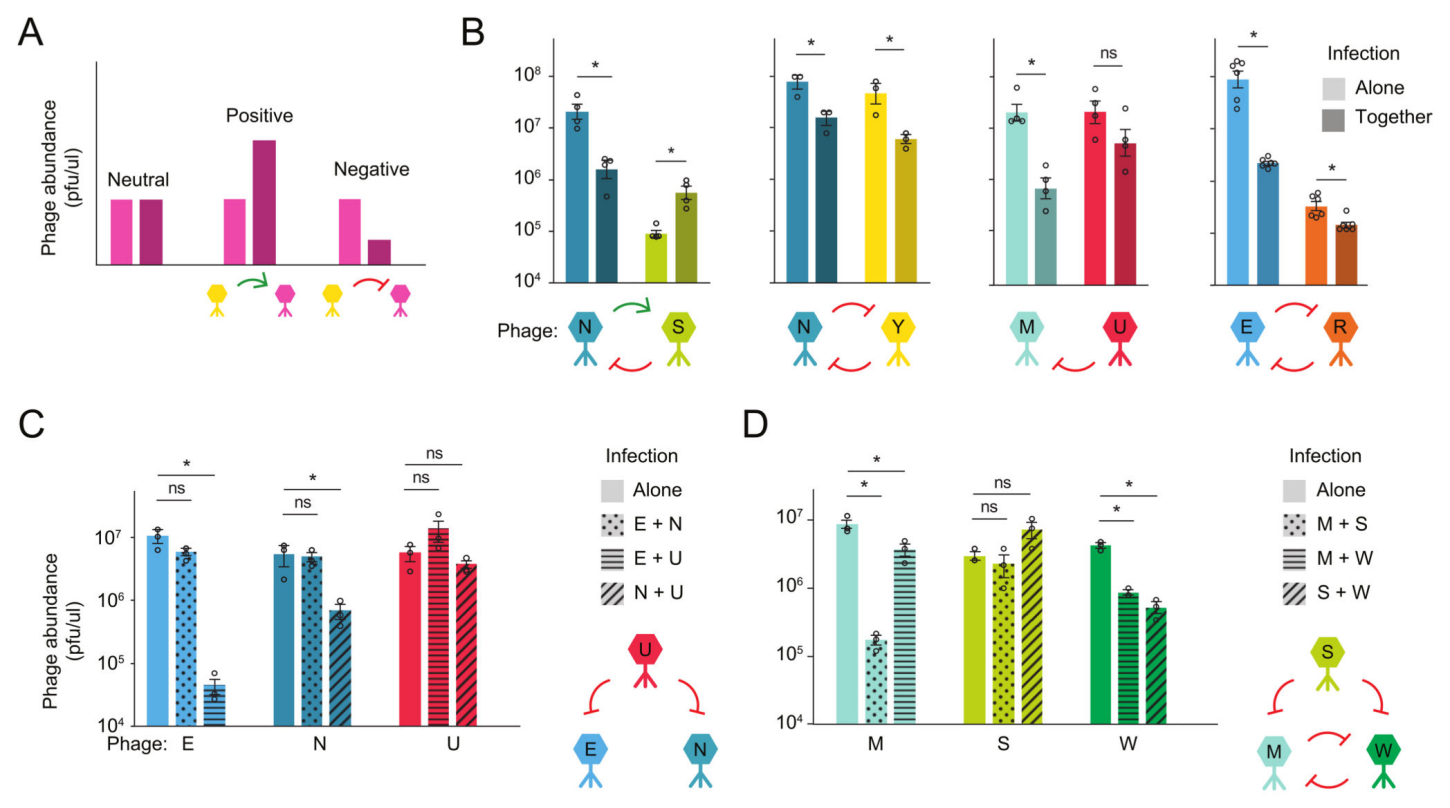
Phage-phage interactions were primarily negative. Pairwise interactions for 2-species and 3-species phage communities. To determine the direction of interactions, we measured a species’ abundance when infecting alone versus when co-cultured with another phage species. All phage samples were passaged for 1-3 days to allow populations to reach equilibrium before measuring each species abundances through top agar plating. Outlined dots represent the abundance of each phage in a competition experiment in each biological replicate (N=3-6 replicates); colored bars are the mean ±SE. For statistical comparisons: ‘ns’ is a non-significant interaction and asterisk indicates significant p<0.05 using a two-sample two-tailed student’s t-test, after adjusting for multiple comparisons using the Benjamini-Hochberg procedure. For each community, we plot a graphical representation of the significant interactions, with green arrows for positive interactions, and red for negative interactions. (**A**) Example results showing how ecological interactions are determined through changes in the pink phage species’ abundance. During coinfection, the yellow phage species either had no impact, increased, or decreased the abundance of the pink phage species, relative to when the pink phage species was infecting alone. We classify these interactions as neutral, positive, or negative, respectively. (**B**) Phage interactions for 2-species communities 2, 3, 6 and 7 (left to right). From left to right, adjusted p-values are: 0.006; 0.004; 0.033; 0.027; 0.003; 0.147; <1e-04; 0.012. (**C**) Same as (B), but showing pairwise interactions between 3-member community 8 and (**D**) community 9. From left to right for (C) and (D), adjusted p-values are: 0.131, <1e-03, 0.968, 0.025, 0.357, 0.182, <1e-03, 0.047, 0.432, 0.089, 0.001, 0.003.

Ecological interactions can be critical to the composition and properties of diverse communities (20). We, therefore, sought to understand how different species interacted i.e., how one species affected the growth and reproduction of other species (Fig. 3A). We determined the direction of interactions for a range of 2-phage (Fig. 3B) and 3-phage (Fig. 3C and D) communities by comparing each species’ abundances when grown in a community compared to when grown alone. This experiment revealed that pairwise interactions were typically negative with examples of both competition (-/-) and amensalism (0/-). None of our phage pairs showed signs of mutualism, but one interaction was asymmetric: phage S benefited from co-culture with phage N, while phage N did worse in co-culture (+/-) indicating an exploitative interaction. The predominance of antagonistic interactions in our communities was consistent with the rapid exclusion of many species early in passaging and with the robustness of our communities to perturbation, as negative interactions can stabilize coexistence (20).

### Coexistence did not depend on resistance evolution

Previous studies have found that the evolution of phage resistant mutants within a bacterial population can promote phage coexistence (9, 10). Despite our attempts to avoid bacterial evolution by filtering between passages, it was possible that bacterial diversification took place over each individual incubation, potentially mediating coexistence. To test this possibility, we investigated whether phage resistant bacterial mutants emerged between our filtering steps i.e. within individual 24-hour passages. We assembled and passaged three biological replicates of three communities from Figure 2B (communities 2, 3, and 6) and then isolated *E. coli* by plating the unfiltered sample at three time points post-infection (4, 8, and 24 hours) during four of the 24 hour passages (passages 1-2, 4-5, 8-9, 11-12). We did not detect colony growth at any passage apart from a single passage for one replicate each of community 2 and 6 (fig. S8). We tested these *E. coli* colonies for phage resistance by top agar plating of phage stocks on cultures grown from three colonies per time point and saw inhibited plaque formation. This indicated that the bacterial population was resistant to both members of their community and thus was unlikely to preferentially support the growth of an individual phage species. In addition, phage diversity persisted in all of the communities throughout the twelve passages, irrespective of the presence or absence of bacterial growth (fig. S8). These data indicated that phage coexistence did not rely on the evolution of phage resistance within the bacterial population.

### Productive coinfection of individual cells was rare

The phage population outnumbered the bacterial cells (i.e., had a high multiplicity of infection, or MOI) at the onset of almost every passage (fig. S9). This suggested that multiple lytic species could be sharing resources within the same coinfected cells, potentially causing interactions between phage species that lead to their coexistence (21–23). To investigate this hypothesis, we tested for productive coinfections by individually sorting coinfected cells using flow cytometry and then identified the different species produced after lysis (24, 25) (fig. S10A and B). For this experiment, we selected a representative community (community 2) consisting of phages N and S. We found that the proportion of individual cells producing both phage N and phage S was negligible (<10%, fig. S10C), and lower than the expected proportion of coinfected cells (92 - 100%, based on the joint Poisson distributions given the MOI for each phage). Productive coinfections were also negligible for cells infected with a different set of coexisting species (fig. S10D). These experiments showed that multiple phage species rarely emerged from the same cell, which indicated that phage coexistence was not supported by productive coinfection of the same bacterial cell (21–23).

### Host phenotypic heterogeneity supported lytic phage coexistence

**Fig. 4:**
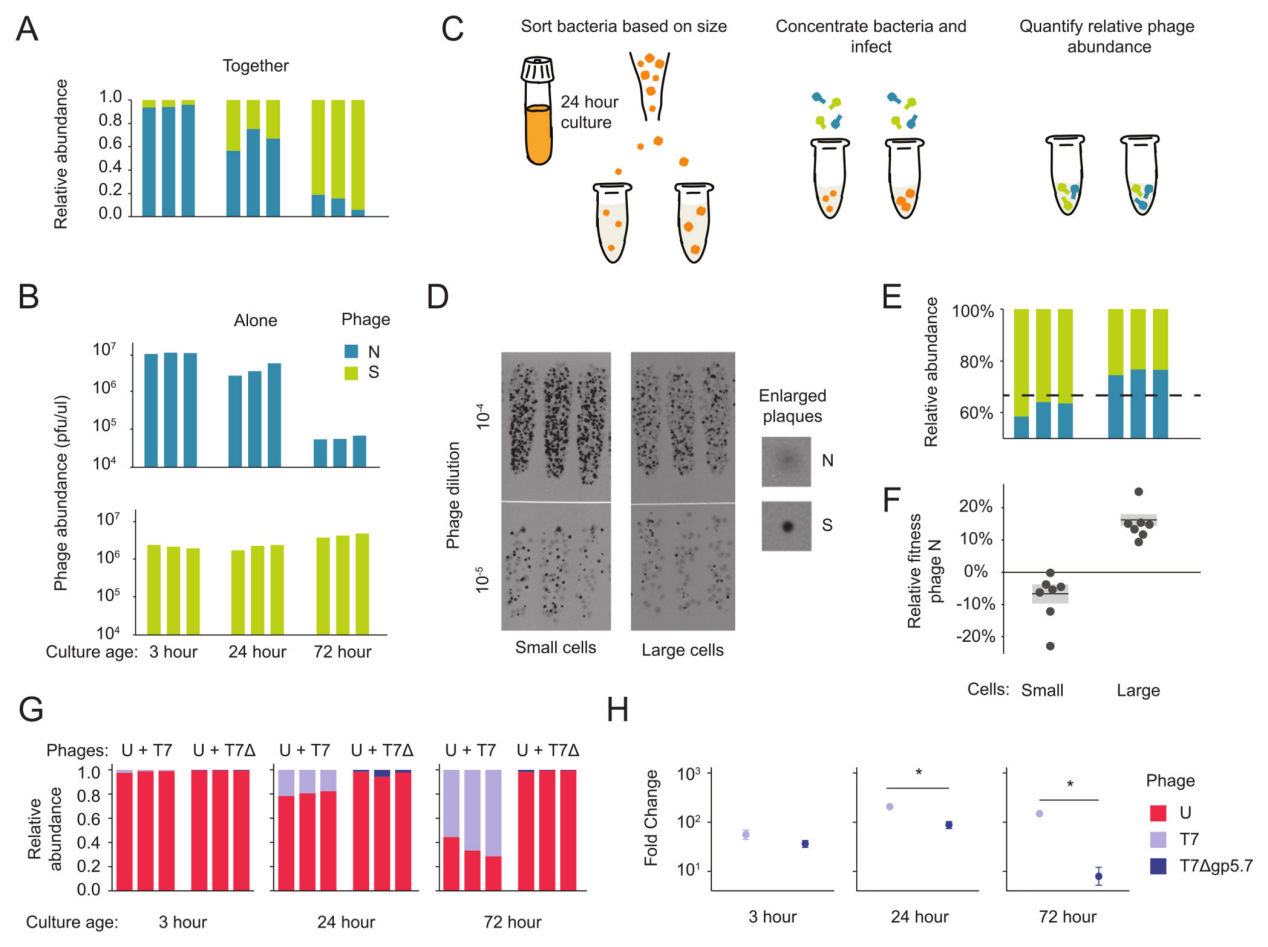
Host phenotypic heterogeneity supported phage diversity. Specifically, a mixture of fast- and slow-growing cells supported phage coexistence. (**A**) We infected bacterial cultures at different ages with community 2 that had been passaged to equilibrium and then determined the relative abundance of each phage species through plaquing. Culture age determined which species was dominant: In the 3-hour culture, phage N was dominant; in the 24-hour culture, the proportions were relatively equal; and in the 72-hour culture, phage N was in the minority [95% confidence intervals = 0.91 to 0.97, 0.53 to 0.64, and 0.15 to 0.28, respectively]. Shifting from the youngest culture (3-hour) to the oldest (72-hour) therefore reversed the dominant species (P < 2 x 10−16; two-sample, two-tailed z test for equality of proportions) (n = 3). (**B**) Infections with phage N or phage S alone qualitatively agreed with the pattern in (A). Phage N achieved a higher abundance in the 3-hour versus the 24- or 72-hour cultures (P = 0.005 and P < 1 x 10−6, respectively, with one- tailed student’s t tests using the Benjamini-Hochberg correction for multiple comparisons) and a higher abundance in the 24-hour versus the 72-hour culture (P < 1 x 10−6). By contrast, phage S achieved a higher abundance on the 72-hour versus both the 24- and 3-hour cultures (P = 0.004 and 0.002, respectively). Phage S achieved a similar abundance in the younger cultures (3-hour versus 24-hour) (P = 0.56) (n = 3). (**C**) Schematic for determining the relative fitness of a phage species on small and large cells from the same 24-hour E. coli culture: Cells were separated by size, reconcentrated, and infected with community 2. Each species’ abundance was determined after incubation through top agar plating. (**D**) Phage N and S abundances after infecting small and large subpopulations (n = 3). (**E**) The relative abundance of each phage species from (D) with the dotted line indicating the starting proportion of phage N. (**F**) Phage N decreased in relative abundance on small cells and increased on large cells (P = 0.033, P < 1 x 10−4, respectively, using a one-tailed one-sided t test, null hypothesis true mean = 0). The relative fitness of phage N is its final relative abundance divided by its starting relative abundance of 0.66. Gray dots indicate n = 7 total replicates from (D) and (E) and an additional flow cytometry run (fig. S17). The gray-shaded regions indicate ± SE and are bisected by a black line indicating the mean. (**G**) To investigate the molecular basis of coexistence, we coinfected cultures of different ages with phage U and either phage T7 or T7Dgp5.7 (i.e., T7D). T7 generally reached a higher abundance than phage U after coinfection (fig. S19A), so we added T7 and T7Dgp5.7 at a lower MOI (0.5) than phage U (5.0) to more easily detect growth differences. Each bar plot shows the final relative abundance of each phage after 24 hours of incubation (n = 3). (**H**) We calculated the log10 fold change in absolute abundance of phage T7 or T7Dgp5.7, in competition with phage U, on different cultures from (G). Each dot reflects the log10 fold change relative to each species’ starting abundance ± SE (n = 3). When in community with phage U, T7Dgp5.7 did worse than T7 on the older cultures (24-hour culture: P = 0.003; 72-hour culture: P = 0.001, using one-tailed t test), but there was no significant difference on the 3-hour culture, suggesting that the phage protein Gp5.7 is important in competition for slower-growing cells. Asterisks indicate significant comparisons.

Given that each phage species infected different cells within the population (fig. S10), we tested whether coexistence emerged because different lytic species were preferentially infecting phenotypically different cells. The host populations in our experiments were isogenic, but cells within a genetically clonal population can still differ from one another phenotypically by adopting different physiological states (26). Virtually all the bacterial cells in a community were immediately infected at the onset of each passage (figs. S9 and S10) and rapidly lysed (fig. S11), which suggested that coexistence relied on pre-existing host heterogeneity rather than host phenotypic changes throughout the passage. One well-documented form of phenotypic heterogeneity among our normal host population (a 24 hour old culture) is that cells differ in how quickly they start regrowing when diluted into fresh media (27, 28). Further, bacterial growth state can affect the reproduction of different phage species (29, 30). As such, phage coexistence could be supported by phenotypic heterogeneity if such cell-to-cell differences resulted in differential production of each phage species. We again chose community 2 as a representative community to test this hypothesis (Fig. 4A-F), since it showed a consistent composition over time (Fig. 2B, fig. S12).

We first tested whether changes to growth potential of the entire culture affected relative abundance of each phage species. To do this we infected 3 hour, 24 hour, or 72 hour cultures of *E. coli* since cultures younger or older than 24 hours have faster or slower regrowth, respectively (fig. S13) (28, 31). Consistent with our hypothesis, we found that changing the culture age completely reversed which species was dominant in community 2. As the age of the culture increased from 3 to 24 to 72 hours the community composition flipped from an average of 95% to 59% to 21% phage N, with a similar pattern in a replicate experiment (Fig. 4A, fig. S14). Infecting these cultures with each of the two species individually, as single-phage monocultures (Fig. 4B, fig. S15), showed a similar pattern as coinfection (Fig. 4A, fig. S14). Overall, these results suggest that fast growing bacteria preferentially produce phage N while slow growing bacteria preferentially produced phage S.

We thus propose that the observed fitness differences of each phage species on younger versus older *E. coli* cultures reflect fitness differences on different growth phenotypes within a single *E. coli* population. To test this hypothesis, we separated the slow and fast growing *E. coli* subpopulations from a 24 hour culture by sized-based flow cytometry sorting, since smaller sized cells from an overnight culture regrow more slowly than larger cells (fig. S16) (28, 32). We sorted and infected 20,000 small or large sized cells with phage community 2 and then measured phage abundances using plaque morphologies (Fig. 4C and Methods). Results from two independent flow cytometry experiments (7 total biological replicates) showed that each phage species did exhibit a fitness advantage on a different growth phenotype (Fig. 4D-F, fig. S17), consistent with our previous culture-level findings (Figs. 4A and B). Phage N, which had an advantage on younger, fast-growing cultures (3 hour), also had a fitness advantage on the large, fast growing cells (p = 0.033, N=7, one-sample, one-tailed t-test compared to baseline relative abundance = 0.66, mean increase = 16.23%) (Fig. 4F). Conversely, phage N had a disadvantage on the slow growing culture (72 hour) and on the small, slow growing cells (*p*<1e-04, N=7, one-sample, one-tailed t-test vs. baseline abundance = 0.66, mean decrease = 6.71%). These results show that even within a clonal host population, there is sufficient phenotypic heterogeneity to allow niche separation of phage species from community 2, thus allowing coexistence between taxonomically diverse phage species.

### Phage-encoded protein controlled T7 community abundance

What are the molecular mechanisms that drive this niche separation? To explore this, we turned to the well-characterized model phage, T7. This phage reproduces well on slow-growing *E. coli* cells due to the phage-encoded protein Gp5.7 which inhibits the host-encoded RNA polymerase *RpoS* that is expressed during slow growth (fig. S18) (29). Therefore, we reasoned that *gp5.7* might help T7 compete for slow growing cells when in community with another phage. To test this hypothesis, we first generated T7-based communities (Methods) and found that T7 and phage U persisted together across passages. We confirmed that this community was ecologically stable (fig. S19) and found amensalism between T7 and phage U (-/0, fig. S20), as was common in our previous experimental communities (Fig. 3). We next investigated the role of *gp5.7* in T7’s ability to compete for slow growing cells by coinfecting cultures of different ages with phage U and either wild-type T7 or T7 with a *gp5.7*, deletion, T7Δ*gp5.7*. When in competition with phage U, wild-type T7 performed better on older cultures than on the 3 hour culture (Fig. 4G). However, T7Δ*gp5.7* performed significantly worse than wild-type T7 on older cultures when in community with phage U (Fig. 4G, H), while its replication on the 3 hour culture was unaffected. These experiments, therefore, support the hypothesis that *gp5.7* is important for T7’s differential success on slow growing cells when in community with another phage. Further experimentation would be necessary to evaluate whether *gp5.7* contributed to T7’s specialization on the slow-growing subpopulation of *E. coli* cells in a manner that allows for its coexistence in community with phage U. The 27 phage species from our collection lack a homologue of *gp5.7* so we expect that diverse molecular mechanisms could allow different phage species to specialize on different bacterial growth states.

## Discussion

We have discovered that multiple lytic phage species can stably coexist while infecting a single clonal *E. coli* host population. At least two distinct phage species coexisted within all 46 synthetic communities that we constructed, and there were no communities in which one phage species excluded all others. This coexistence was maintained over 12 passages, and always included multiple lytic species from distinct families (Fig. 1E and fig. S4). These phage communities were ecologically stable, whereby the coexisting species grew in frequency when they were initially a rare proportion of the population (Fig. 2). Ecological interactions within these communities, as in many other microbial systems (20, 33), were largely negative (Fig. 3). In spite of competition, this coexistence was explained by niche separation: each species within community 2 persisted by preferentially replicating on cells with different growth phenotypes that spontaneously arose within the clonal *E. coli* host population (Fig. 4).

In contrast with the typical assumption that phage diversity requires either host genetic diversity or spatial separation (8, 13), we found the stable coexistence of lytic species on a well-mixed, genetically homogeneous *E. coli* strain. These findings are consistent with studies in other microbial systems where, given enough starting diversity, multiple competing bacterial species can coexist, even when cultured on a single growth-limiting resource (34). We should therefore expect the coexistence of multiple phage species even in the simplest possible environments. Stable communities of phages that arise on a shared host population would be hotspots for driving the specialization, recombination, and social interactions between diverse phage species (35).

Phenotypic variation in host physiology allowed for the coexistence of different phage species in a representative community, and manipulating this host phenotype allowed us to change the relative abundance of different species (Fig. 4). We expect that phenotypic heterogeneity will be broadly relevant for understanding phage coexistence. Even within clonal lab-grown *E. coli* populations, phenotypic heterogeneity arises spontaneously through molecular fluctuations in transcription and translation and is therefore unavoidable (26). Higher levels of cell-cell heterogeneity are expected in natural settings (36) where a broad range of sequencing studies have previously found that phage diversity exceeds host diversity (7, 37–40). Thus, we expect that the degree of phage diversity supported by a single bacterial genotype is likely to be greater in nature than we found in this study.

Just as multicellular organisms host a wide array of bacterial species within their microbiome, our results show that a single bacterial strain can, itself, host a diverse community of phage species. The ecological frameworks used to understand competitive coexistence in more complex organisms served as our guide for characterizing how factors, including life history traits, ecological interactions, and resource partitioning led to stable phage coexistence and the assembly of our phage communities (15, 41, 42). Our results show how even under extremely restrictive, competitive conditions, the simplest biological entities on Earth still found paths to coexistence.

## Materials and Methods

### Bacterial culturing

*E. coli* strain BW25113 was used for all experiments with incubations at 37°C. Culturing of uninfected bacteria was done in 3 ml of LB broth (Miller) (American Bio) in a 14 ml snap-cap tube (Falcon) grown with shaking for 24 hours unless otherwise noted. Frozen stocks of *E. coli* were made with 40% glycerol (final concentration) and kept at -80°C. To inoculate the culture we used a single colony of *E. coli* from an LB agar plate that had been streaked 24 hours before the start of a passaging experiment. This plate was then stored at 4 °C and a single colony was used as inoculum for the remainder of the experiment. Colonies were used as inoculum for experiments on community assembly experiments, stability tests, and interaction experiments (Figs. 1-3).

Previous work has shown that the type of inoculum used for an overnight *E. coli* culture can change the consistency of phage titers and the consistency of bacterial phenotypes in the culture (44, 45), with the colony inoculum giving more variability than using a frozen inoculum. To reduce the variability in the phage titers throughout passaging, we used the frozen *E. coli* stock as a starting inoculum for subsequent experiments (Fig. 4). Passaging on cultures started from a frozen stock showed more stability to community 2 (fig. S12) than passaging on bacteria started from a colony inoculum (Fig. 2). See the phage infection section for more detail.

### Environmental phage isolation

Individual phage species were isolated from different environmental samples collected between September and October 2020 from New Haven (Connecticut, USA). Each environmental sample was collected in a sterile 1.5 ml tube and briefly vortexed. To increase the abundance of *E. coli* phages, 50 μl of an overnight culture and 500 μl of LB + salts were mixed with each sample (46). After an overnight incubation without shaking the supernatant from each sample was then filtered to remove the bacterial component. The filtrate was plated on a top agar plate and samples that generated a transparent spot on the lawn of *E. coli* were then used for two rounds of single plaque purification. Single plaques from the last round of purification were then used to make permanent stocks of each phage species and subjected to genome sequencing. Out of 172 environmental samples collected we were able to single plaque purify phage species from 77 samples. We sequenced 48 samples, excluding those samples that showed multiple plaque morphologies or samples where the permanent phage stock had degraded or had a low titer. We chose 27 phage species for inclusion in our collection. We excluded sequenced samples from our collection for the following reasons: sequencing failure, genome assembly produced multiple contigs, or if the phage species had >99% sequence homology to another phage species in the collection. Each phage species in our collection was given a shorthand name used in the paper as well as a formal name (Data table S1) according to the rules of the International Committee on the Taxonomy of Viruses (ICTV) (16).

### Bacteriophage stock handling

High-titer stocks of each phage species were generated by mixing a single plaque with a 24-hour culture that was diluted 1:20 into 600 μl LB + salts. After 24-hour incubation at 37°C, the phage stock was passed through a 0.22 μm filter to remove any surviving bacteria. Each stock was titered by plating 10-fold serial dilutions on a top agar lawn of *E. coli* and quantifying plaques. These working stocks were stored at 4 °C and discarded after 2-3 weeks. Permanent phage stocks were frozen with 40% glycerol (final concentration) and stored at -80 °C.

### Top agar preparation and plaque quantification

We visualized plaques of each phage species for quantification and isolation by using the double-agar overlay method. The bottom layer was comprised of LB plates made with molecular-biology grade agarose since higher quality agarose, as compared to agar, gave a smoother surface for plating phages. The top agar was made of LB + salts (final concentration 100 mM CaCl_2_, 100 mM MgCl_2_, 0.8% w/v agarose, 5% glycerol) and was stored at 55 °C. Glycerol was added to improve plaque visualization (47).

For each plate we mixed 6 ml of top agar and 150 μl of a 24-hour *E. coli* culture and poured onto the surface of the LB agarose plate. Serial dilutions of phages were done in LB + salt and then plated by spotting or dripping a small volume onto the surface of the top agar. We typically plated 5 μl – 10 μl of 10-fold dilutions from 10^-3^ to 10^-8^. After overnight incubation, plates were scanned using the Epson Perfection V850 Pro Scanner at 800 dpi using the transparency setting. Scanned images were then imported into Adobe Illustrator to quantify and visually distinguish plaque morphologies of different phage species. For some experiments we changed the top agar recipe to enhance plaque visualization. To increase the plaque size of phage R we omitted the glycerol in the top agar, doubled the volume of overnight culture used (300 μl), and reduced the concentration of agarose in the top agar to 0.4% w/v. The plaque morphology of some phage species in our collection was variable between different working stocks or throughout passaging experiments, so we routinely verified the identity of these species using whole genome sequencing.

T7 generated much larger plaques than the other phage species in our collection, so we quantified the phage species in T7 communities by top agar plating on both *E. coli* BW25113 and on a mutant *E. coli* strain resistant to T7. This bacterial mutant was isolated by picking a single colony that grew on a top agar lawn of *E. coli* BW25113 infected by T7. We validated that this mutant was only resistant to T7 and not any of the other phage species by top agar plating serial dilutions of individual phage stocks. To quantify phage N in community 2 for fig. S14 B-D, we plated on a bacterial strain resistant to phage S that was isolated using the same technique.

### Bacteriophage DNA sequencing and computational analysis

#### DNA extraction and deep sequencing

Genomic DNA from 100 μl of individual phage species stocks or 50 μl of phage communities was purified using the ZR-96 Clean and Concentrator Kit (Zymo) according to the manufacturer’s instructions. We excluded the DNase or protease treatments that are sometimes suggested for phage DNA extraction (46, 48). Each sample was sent to SeqCenter, a sequencing laboratory located in Pittsburgh, PA, for individual library preparation and short-read sequencing using their Illumina Whole Genome Sequencing 200Mbp service. At SeqCenter each sample was prepared for sequencing using tagmentation-based Illumina DNA library preparation and IDT 10bp UDI indices. Sequencing was performed on the Illumina NextSeq 2000 which generated 2 x 151 basepair reads. SeqCenter performed demultiplexing, quality control, and adapter trimming using bcl-convert version 3.9.3 (proprietary Illumina software). After these steps, the average total reads from the paired sequencing (R1+R2) for each sample was 2.6 million base pairs and >92% of base pairs in each sample had a quality score of Q30. The high-quality fasta files provided by SeqCenter were used for downstream analysis.

#### Isolated phage species genome assembly

To generate full genomes from isolated phage species we first trimmed the fastq files produced by the Illumina sequencing using Sickle (https://github.com/najoshi/sickle). Full genome-sized contigs were then assembled using SPAdes version 3.15.0 (49) with default parameters, but using only the first 400,000 lines of the fasta files, since excessive coverage leads to problems with assembly (50). We verified the assembly of a single contig through Bandage and got an average of 40X coverage per phage genome. Individual genomes can be found deposited at NCBI Genbank under accession numbers PP925821-PP925847 (see also Data table S1).

#### Pairwise genome comparisons and lifestyle characterization

Pairwise genome comparisons were performed using the default settings on Virdic (51) on the web application (http://rhea.icbm.uni-oldenburg.de/VIRIDIC/) by submitting genome sequences assembled through our pipeline. Each species was assigned as lytic or temperate by combining bioinformatic and experimental analyses. For bioinformatic analysis of their genome sequences we used two tools: PhaBOX (the PhaTYP (52) module) (https://phage.ee.cityu.edu.hk/) web application and with the classifier BACPHLIP (53). The predictions from these tools (Data table S1) agreed with experimental assays looking at spot turbidity and growth curves of infected cells (fig. S2). We determined spot turbidity by plating a concentrated phage stock on a lawn of *E. coli*.

#### Taxonomy and phylogenetic tree

The phylogenetic tree was generated using VICTOR (https://ggdc.dsmz.de/victor.php) (54) with the formula D0 producing an average support of 65%. See further information and additional trees in our deposited data (43). To identify the taxonomic classification of each phage species we first performed whole-genome blastn searches against the virus database (https://blast.ncbi.nlm.nih.gov/Blast.cgi) to find the genus and family of phages with high identity. Additional taxonomies were identified using PhaGCN (https://phage.ee.cityu.edu.hk/) (55) and the aforementioned VICTOR analysis. To classify phage species in the same genus we used a cut-off of 70% nucleotide identity, respectively, across the full genome length (56).

#### Phage community sequencing analysis

To identify each phage species from a community we first assembled the Illumina sequencing reads into individual contigs as described for isolated phage genomes, except using the whole fasta file to ensure coverage of rarer phage species. We used a local NCBI blastn search (word size of 1000) (57) to compare each community contig against a local database with our collection of 27 phage genomes. The local database was generated by using the makeblastdb BLAST command. We used the average coverage of each contig in the community to calculate the relative abundance of each phage species in the community. None of our phage communities showed evidence of cross contamination from other communities. The most abundant phage species in a community had 1,000 − 4,000 fold coverage of its entire genome. Our limit of detection was ∼ 0.1% of the total phage population or ∼ 1 fold coverage of the least abundant phage’s entire genome.

The *Straboviridae* phage species from our collection had high homology with one another so we used an additional sequencing analysis step to identify each individual species in communities with multiple *Straboviridae* phage species in the starting mixture. We first performed the genome assembly and blastn steps as previously described. This analysis identified the most abundant *Straboviridae* species’ genome as a single contig. The lower abundance species were present as smaller contigs (100-1000 base pairs) containing only the regions of non-homology with the more abundant *Straboviridae* and were identified using a using NCBI blastn (word size of 100) local database containing a unique sequence from each phage species in the *Straboviridae* family. We also used this process to identify *Stephanstirmvirinae* species. A detailed description of this code and the sequences of assembled genomes are deposited on Dryad (43).

### Phage infection experiments

All phage infection experiments were carried out as follows, unless otherwise noted. At the onset of passaging on day 0 the starting phage communities were prepared by using freshly made and titered working stocks of each species. A master mix was assembled for each community (containing 1.2 * 10^6^ pfu of each phage species) and then 10 μl (containing 10^5^ pfu of each phage species) was added to each biological replicate community. For stability experiments (Fig. 2 and fig. S19), the starting communities contained a total of 2 x 10^5^ pfu, with 1% to 99% of each species depending on the condition.

Phage was added to 300 μl LB + salts (final concentration 100 mM CaCl_2_ and 100 mM MgCl_2_) in 2 ml 96-deep-well plates (Celltreat) sealed with piercible aluminum seal (Thomas Scientific). To avoid cross contamination, samples were separated by two wells in every direction if they contained different communities or phage species. A fixed volume or concentration of a 24 hour *E. coli* culture (see next paragraph and bacterial culturing methods for more details) was then added to each well and the plate was incubated at 37°C. After 24 hours the phage was collected by puncturing the foil seal with pipette tips and filtering 100 μl through a 96-deep-well 0.22 μm filter plate (Acroprep). We then used 10 μl of the filtered phage sample to infect the next pasage (dilution of 1:30). The remaining filtered sample was used for top agar plating and stored at -80°C for downstream DNA extraction. For some experimental assays (ie: phage interactions, growth curves), communities were passaged for 1-3 days to allow the composition to stabilize before performing the assay.

We used a colony or a frozen stock as inoculum for overnight cultures of *E. coli* (see bacterial culturing for more details). We added 3 μl to each well for infections using a culture started from a colony. This is equivalent to 4 x 10^4^ colony forming units (cfu)/μl (final concentration) at an OD600 of 5.0, where an OD of 1.0 is 8 x 10^6^ cfu/μl. We added 1.2 x 10^7^ cfu to each well (final concentration 4 x 10^4^ cfu/μl) based on the OD600 for infections using a culture started from a frozen stock. In general, we found that a 24 hour *E. coli* culture had an OD between 4.5 and 6.5.

#### Community assembly

The starting composition of 10-species communities was randomly generated, while the starting composition for 5-species communities was selected semi-randomly to include at least 2 lytic species. To generate 2-species communities with phage T7, we incubated T7 in triplicate with either phage M, N, U, or Y or with the model phages T4 or T5. After 12 passages, T7 was abundant in all communities while only phage U and T5 were still detectable in all biological replicates of those communities.

#### Growth curves

Growth curves were performed in triplicate using 96-well plates with 150 μl LB + salts in each well with 4 x 10^4^ cfu/μl (final concentration) of an overnight culture unless otherwise noted. The OD600 was measured every 10 minutes after infection for 24 hours using an automatic plate reader maintaining a temperature of 37°C. For curves of individual phage isolates (fig. S2), each well was infected at an MOI of 1. For infections using communities 2, 3, 6 and 7 (fig. S11), the phages were first passaged for 2 days to reach stable equilibrium. Bacterial growth was then measured after infecting bacteria with 5 μl of phage communities which is the same dilution factor (1:30) used during passaging. The curves were plotted using the gcplyr package in R (58).

#### Culture age assay

To generate the 3 hour culture used for infections in Figure 4, a 24 hour culture was diluted 1:2000 in 20 ml LB and grown for 3-4 hours to an OD of 0.1 - 0.3. This culture was then pelleted by centrifugation (10krpm for 3 minutes) and resuspended to an OD of ∼ 5.0. The 72 hour culture was generated by inoculating a culture 2-3 days before the infection, while the 24-hour culture was started as previously described. We validated that the OD600 gave an accurate measurement for cell number by quantifying cfu of each culture condition. Each culture was diluted into fresh LB + salts at 4 x 10^4^ cfu/μl final concentration. For infections with a single phage, we used 10 μl of each phage stock or passaged the phage for 1-3 days to allow the phage to equilibrate. For infections with multiple phages, we passaged the community for 1-3 days to reach equilibrium before infecting cells with 10 μl. For infections with T7 or T7Δ*gp5.7* we used 2 x 10^4^ pfu/μl (final concentration) of these phages and 2 x 10^5^ pfu (final concentration) of phage U. Phages were quantified after incubation through top agar plating.

#### Statistical testing

For comparing absolute phage abundances, we log10-transformed phage abundance values and then conducted student’s t-tests, after using the F-test to check for homogeneity of variance between comparison pairs. When conducting multiple tests (Fig. 3; Fig. 4B), we used the Benjamini-Hochberg procedure for false discovery rate (59), and we report adjusted p-values. For comparing phage relative abundances, we determined the number of plaques corresponding to each phage species, pooled across all replicates at the same dilution, and then calculated 95% Binomial confidence intervals using the Agresti-Coull method (60). We conducted statistical tests using R version 4.2.0 (2022-04-22) (61) and using tidyverse version 2.0.0 (62).

### Phage resistance

#### Basal phage resistance rate

To determine whether phage-resistant bacteria were contributing to phage coexistence we first measured the rate of spontaneous mutants within the overnight culture that are resistant to individual phage species using top agar assays (as described above) with the following modifications. We mixed and plated 25 μl of an overnight *E. coli* culture with 40 μl of each phage species stock. After an overnight incubation we quantified the phage resistant cfu by counting the surviving colonies (fig. S21). These mutants were extremely low abundance (∼1 in 10^6^ cfu) which meant that only ∼10 bacteria would be present in each 300ul well containing 1.2 x 10^7^ cfu at the onset of each passage during our experiments. Phage abundances at the end of the passage were typically >10^7^ per ul (Fig. 2) i.e. 10^9^ total pfu per well, which was ∼ 8 logs lower than the resistant population. Thus these resistant bacteria did not seem to explain the coexistence we observed. In addition, cultures infected with more than one phage (fig. S11) did not show evidence of regrowth. This indicated that phage resistant mutants were not reaching high abundance during coinfection and, again, indicated that resistant bacteria were not important for our observations of coexistence. However, to further clarify the role of phage resistance in phage coexistence, we measured the *E. coli* abundance within individual passages (see below).

#### Within-passage phage resistance

Although bacteria were filtered between each passage it was possible that phage resistant bacterial mutants were growing within an individual 24 hour passage, below the limit of detection for a growth curve (fig. S11), and altering phage coexistence. To test for the presence of these bacteria we recreated and passaged three biological replicates of three 2-phage communities (communities 2, 3, and 6 from Fig. 2) and quantified each phage species during pasaging using top agar. We isolated *E. coli* by plating 5 μl of the unfiltered community on LB plates, as well as serial dilutions of the unfiltered sample, at three time points post-infection (4 hours, 8 hours, and 24 hours) for four of the 24-hour passages (between passages 1-2, 4-5, 8-9 and 11-12). We did not detect any *E. coli* colony formation at most time points (our limit of detection was 60 cfu per community). In the two instances where *E. coli* colonies grew, we cultured three colonies per time point to test for phage resistance. We spotted serial dilutions of individual stocks of phage species (phage N and S for community 2 or phage M and U for community 6) on top agar lawns of these cultures. Phage resistance was indicated by inhibited plaque formation in comparison to plating on the parental *E.coli* strain.

### Flow cytometry

#### Flow cytometer settings

The FACS Aria III cell sorter with a 100 μm nozzle was used for all flow cytometry experiments. Events were detected using forward scatter and side scatter triggers and data was obtained in logarithmic mode then analyzed with BD FACS software. The forward and side scatter settings (FSC at 6 volts, SSC at 300 volts) were chosen to avoid background noise from the buffer solution. Cultures were diluted 1:100 in PBS or LB + salt before sorting to ensure accurate cell separation. The PBS added to samples before sorting was passed through a 0.22 μm filter to reduce micro-particles in the buffer that could clog the machine or contribute to background noise.

#### Coinfected single-cell sorting

To determine whether coinfected cells were producing multiple phage species, cells were infected at various MOIs with phages from community 2 or 6 and incubated for 1.5 hours before sorting, which allowed time for phages to adsorb, but before the phages caused lysis (fig. S11). Cells were gated to select for the entire bacterial population and then single-cell sorted into individual wells of a 96-well PCR plate containing 20 μl of LB + salt. Immediately after sorting the plate was sealed with pierceable aluminum foil and moved to 37C for 24h incubation. To determine the phages present, 5 μl of each well was plated for the top agar assay.

A number of control experiments were performed to verify the suitability of this technique for determining the phage produced by individual cells. To verify that extracellular phages were not present in the sorted drop together with a dispensed cell, we individually sorted the background particles (with the same forward and side scatter as *E. coli*) that were present in a sample containing unfiltered LB with the same volume of phage as we used during infection. We then checked for any plaques by plating using the top agar assay. Out of 120 wells we found 1 plaque in 3 different wells which confirmed that our error rate for counting free virus (instead of phage burst from a cell) was very low (1 plaque per 40 wells).

We then tested the sorting accuracy for dispensing single bacterial cells by sorting an uninfected culture and plating the entire well on an LB plate. After overnight incubation of the agar plate, we found only 1 well out of 72 wells that grew 2 colonies (none grew more than 2 colonies) while all the rest grew either 0 or 1 colonies (fig. S10A). This indicated that the flow cytometer was accurately sorting individual cells.

#### Size-based flow sorting and infection

To sort small and large cells, we gated the 24 hour culture’s bacterial population by forward scatter capturing the smallest and largest 30% of cells. For growth curves of small and large cells, we sorted 100 cells from each size-gated population into a well of a 96-well plate containing 200 μl LB. We then proceeded with the growth curve analysis as previously described. We confirmed the accuracy of our sorting by quantifying the cfu through plating of each group immediately after sorting.

For assaying the fitness of each phage species from community 2 on different bacterial subpopulations, we gated and sorted small and large populations using 2-way sorting at 10^4^ events/second to sort a total population of 4 million cells from each gate into an empty 2 ml collection tube. The total volume of sorted cells exceeded the 2 ml maximum volume captured in the collection tube so we periodically emptied the collection tube into a 50 ml tube. Once sorting for both populations completed (∼ 40 minutes) we reconcentrated the bacteria by collecting each sorted population onto a different 0.22 μm syringe disc filter fitted into a filter holder. We used sterile forceps to move the disc filter from the syringe holder into a 1.5 ml tube containing 250 μl LB + salts. The bacteria were released from the filter into the media by briefly vortexing and 5 μl was used for serial dilutions to quantify bacterial abundances, which differed less than 1.3 fold between the small and large sized samples in both experiments. After resuspension, we infected 3-4 biological replicates of small and large sized cells. Each replicate containing ∼10^4^ cfu and ∼9 x 10^4^ total pfu of community 2. The phage sample used for infections was taken from community 2 at passage 3 of fig. S12, because phage N and S showed greater stability in this experiment than in Fig. 2 or fig. S8. The total reaction size was 25 μl per replicate. After 24 hours of incubating the infected samples, we filtered and quantified the abundances of phage N and S through top agar plating.

### T7 *gp5.7* deletion and *gp5.7* homology search

To delete the gene *gp5.7* from phage T7 we used previously described techniques involving homology-based recombination and CRISPR counter selection (63). For the recombination step, we first synthesized plasmid pNCP28 (IDT) which is a pUCIDT-Kan-based vector that contains an insert with homology to the regions flanking *gp5.7* (275bp upstream and 137bp downstream). To generate T7 phages that recombined with the plasmid and deleted *gp5.7*, we then infected 50 μl of an overnight culture of *E. coli* containing pNCP28 with 10 μl of a T7 phage stock. After lysis we counter-selected for the recombined T7 subpopulation by plating on *E. coli* cells with a pCas9 plasmid that targets *gp5.7* (pNCP29). This plasmid was generated using BsaI cloning with primers NP130 and NP131 as previously described (64). The T7 plaques that grew on the pNCP29 lawn were replated. The deletion was verified in these subsequent plaques by amplifying and the region around *gp5.7* using primers NP132 and NP133 and sequencing the product. Primer sequences and the pNCP28 inserted sequence are listed on Data table S2. We determine that the phage species in our collection did not contain homologs to *gp5.7* by running a local NCBI blastn search (word size of 100) comparing the DNA sequence of *gp5.7* with the whole genome sequences of each species in our collection.

## Supplementary Material

Supplementary Material

## Data Availability

Genome sequences for species in our phage collection are deposited at NCBI GenBank under accession numbers PP925821-PP925847 (also see Table S1). The contigs generated from sequencing of phage communities and the code used to analyze the community composition was deposited on Dryad (43), as were the alternate phylogenetic trees generated by the VICTOR tree builder.
