# Supplementary material for: Diverse phage communities are maintained stably on a clonal bacterial host
Source: Science. Author manuscript; Available in PMC 2025 Jan 7. (PMC7617280; doi:10.1126/science.adk1183)
Supplement: Supplementary Material [file EMS202075-supplement-Supplementary_Material.pdf]

Science
AAAS

Supplementary Materials for

Phage community dynamics and ecology on a clonal bacterial host

Nora C. Pyenson[1-3*], Asher Leeks[3,4], Odera Nweke[3], Joshua E. Goldford[5], Jonas Schluter[1, 2], Paul E. Turner[3,4,6,7], Kevin R. Foster[8,9*], Alvaro Sanchez[10*]

*Corresponding authors. Email: alvaro.sanchez@usal.es (AS); kevin.foster@biology.ox.ac.uk (KRF); nora.pyenson@nyulangone.org (NCP)

The PDF file includes:

Materials and Methods
Figs. S1 to S21
Data tables S1 to S2
References (44-64)

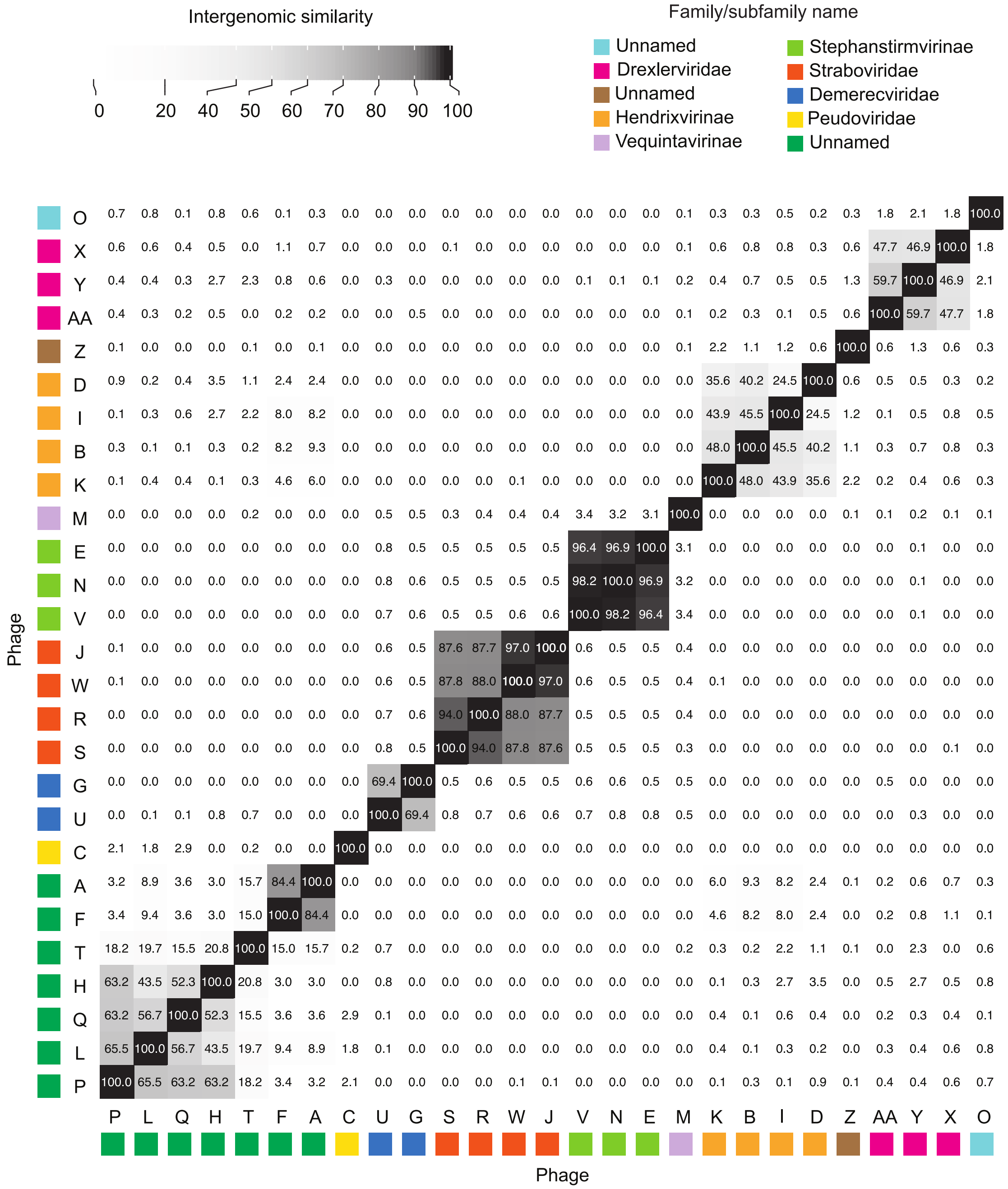

Fig. S1. Phage genomes were unique, but had sequence homology within the same family. We determined the sequence similarity between phage species by performing pairwise comparisons of all 27 genomes in our collection using VIRDIC (*51*). Darker colors indicate higher levels of intergenomic sequence similarity. Boxes below each species names are colored by their family classification, but not all families are named in ICTV (*16*), so subfamily names are listed in the legend in some cases. Species were ordered along the x- and y-axis by sequence similarity, but this ordering naturally produced clustering among species in the same family (ie. phages marked with the same color). Species within the same family are highly divergent from species in different families (average <0.5% similarity in pairwise comparisons).

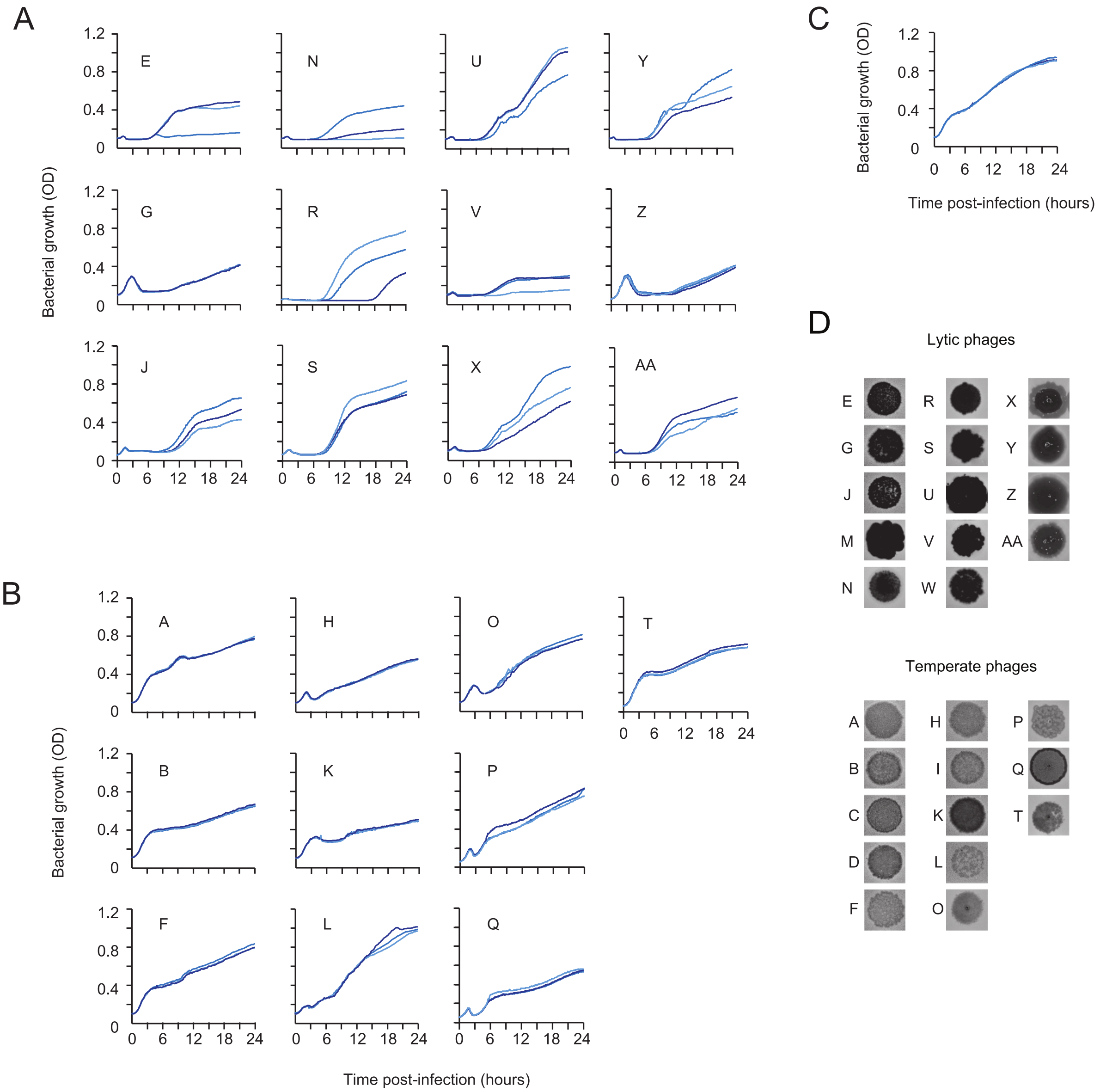

Fig. S2. Growth curves and spot turbidity indicated the phage replication strategy.
(**A**) To validate the lifestyle of the species that were predicted through bioinformatics (Methods, Data table S1) we measured bacterial density (OD 600nm) every 10 minutes for 24 hours after infection for a subset of lytic species (N = 3 biological replicates). Species names are inset within each growth curve. After lysis the OD was reduced to the limit of detection (OD ~ 0.1) and growth remained undetectable for several hours. These features indicated that these were lytic species. Regrowth 4 hours after lysis is likely due to phage-resistant bacterial mutants that have emerged. (**B**) Same as (A) except a subset of temperate species. Apart from phage Q, these species did not reduce the density to the detectable limit at any point during infection. (**C**) Same as (A) except no phage was added (**D**) High titer phage stocks were spotted on a lawn of *E. coli* to additionally validate their lifestyle, since temperate species tend to produce more turbid spots.

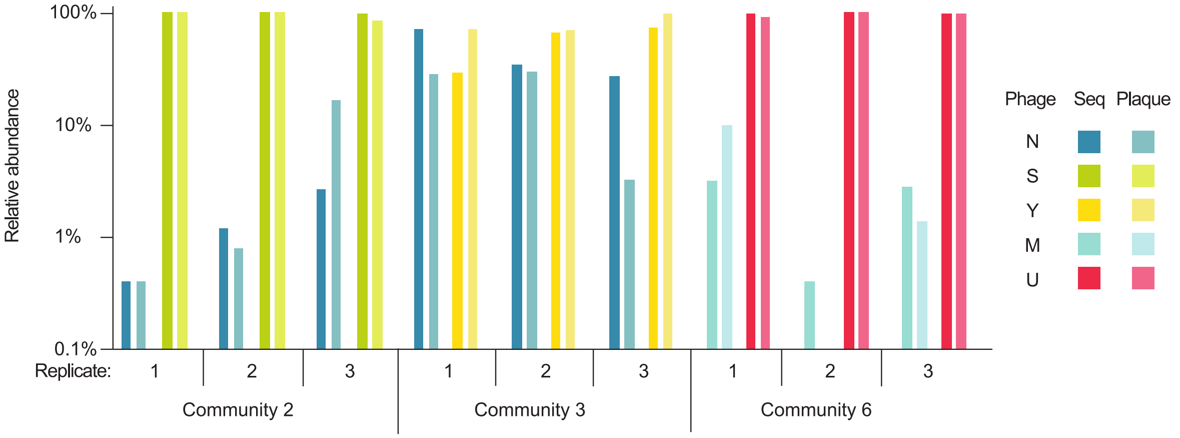

Fig. S3. Plaque counting and deep sequencing gave similar results for the abundance and identity of species in each community.

To validate that our deep sequencing accurately identified the abundance and identity of species in our communities we plated three communities on top agar at the final passage (N = 3 biological replicates). We then determined the relative abundance of each species through plaque morphology (Plaque) and compared these values to the relative abundances calculated through deep sequencing (Seq).

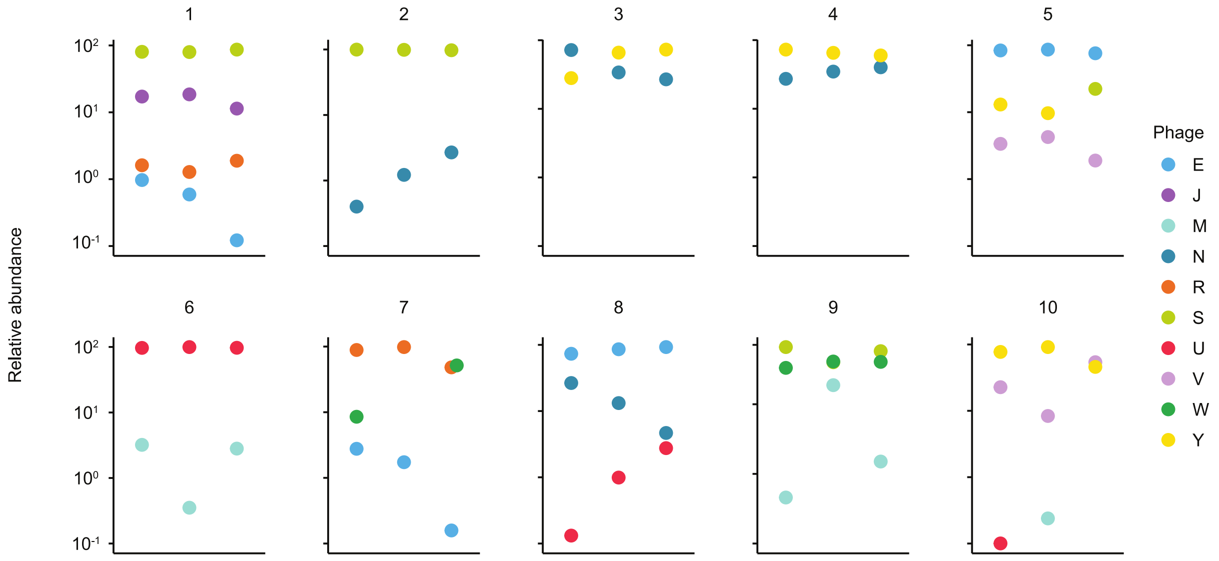

Fig. S4. Relative abundances of species for communities on the log scale.
To compare the species abundances found in Fig. 1E (shown in linear scale) more easily with subsequent figures (shown in log scale), we have replotted the Fig. 1E data on the $\log_{10}$ scale. The relative abundance of each phage species at the final passage was determined through deep sequencing of the phage lysate for each community (numbered above each chart) (N = 3 biological replicates, shown left to right within each plot). The relative abundance of each phage species was calculated based on the average coverage of each phage species' genome.

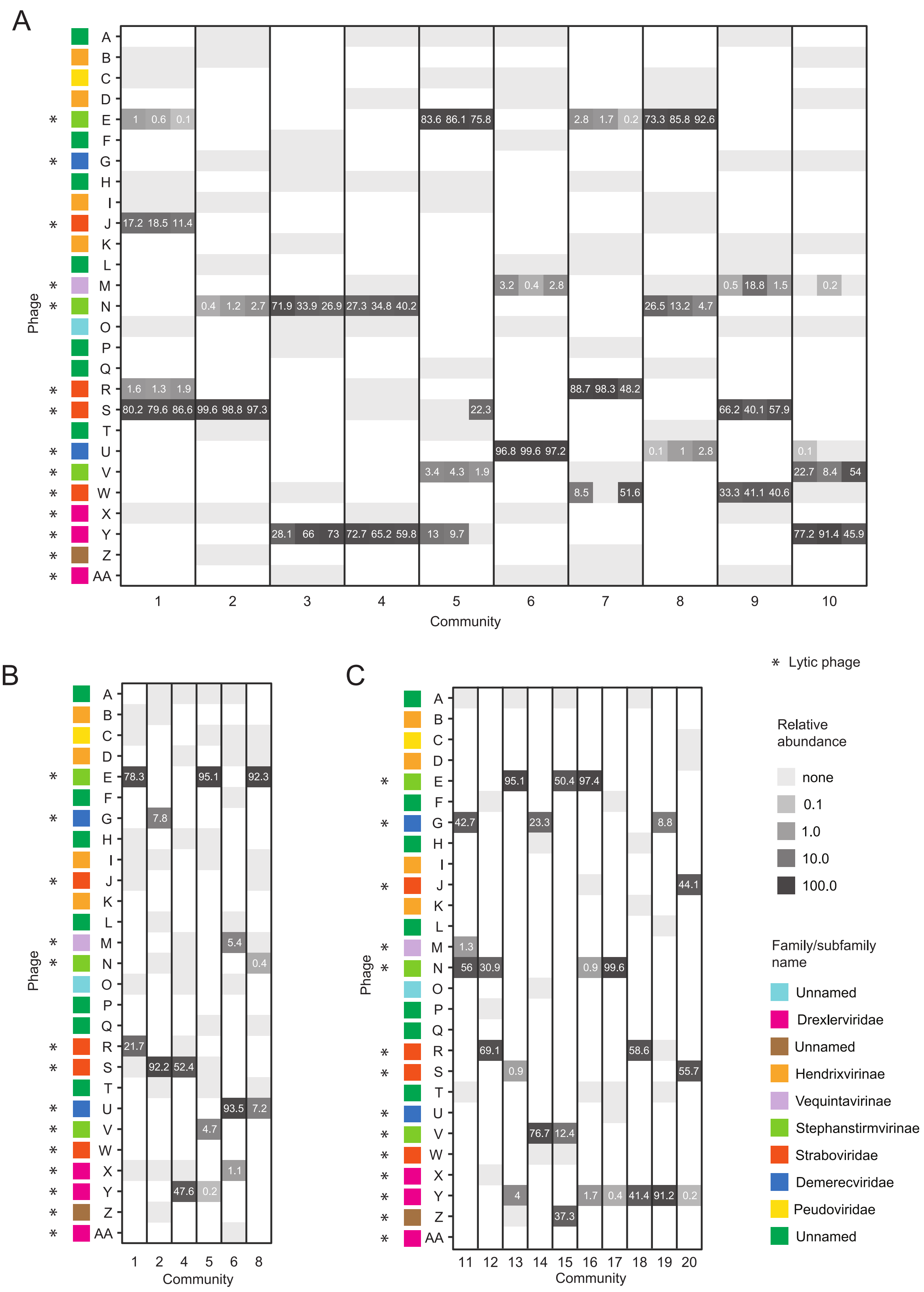
A
B
C
Phage
Community
* Lytic phage
Relative abundance
none
0.1
1.0
10.0
100.0
Family/subfamily name
Unnamed
Drexlerviridae
Unnamed
Hendrixvirinae
Vequintavirinae
Stephanstirmvirinae
Straboviridae
Demerecviridae
Peudoviridae
Unnamed

Fig. S5. Relative abundances for phage species found in all communities and replicates. Deep sequencing of communities at the final passage revealed the relative abundance of each phage species. The relative abundance was calculated based on the average coverage of each phage species' genome. Darker colors indicate higher abundance, with color scaled by log relative abundance. Phages with "none" were added in the initial community but not detected through deep sequencing at the final passage. The limit of detection was ~ 0.1% of the total population. The family and replication strategy of each phage species is indicated next to the species name. Asterisks indicate that a phage species is lytic. (**A**) The relative abundances for the 10 communities shown in Fig. 1E. (N = 3 biological replicates) (**B**) To determine the impact of our passaging protocol on community composition we repeated the experiment in (A) without filtering *E. coli* between each passage. The starting communities used in this experiment were composed of the same phage species as communities 1, 2, 4-6, and 8 used in (A) (N = 1 biological replicate). These final communities all had similar compositions (with some notable differences) as communities passaged with filtering in (A). (**C**) We tested the limits of coexistence by reducing the number of different phage species in the starting community. The same passaging protocol was used as in (A) except the initial communities contained 5 instead of 10 species and only a single replicate was performed for each community. The species in the starting communities for (C) were chosen randomly, but then adjusted to ensure that the community contained at least 2 lytic species (N = 1 biological replicate).

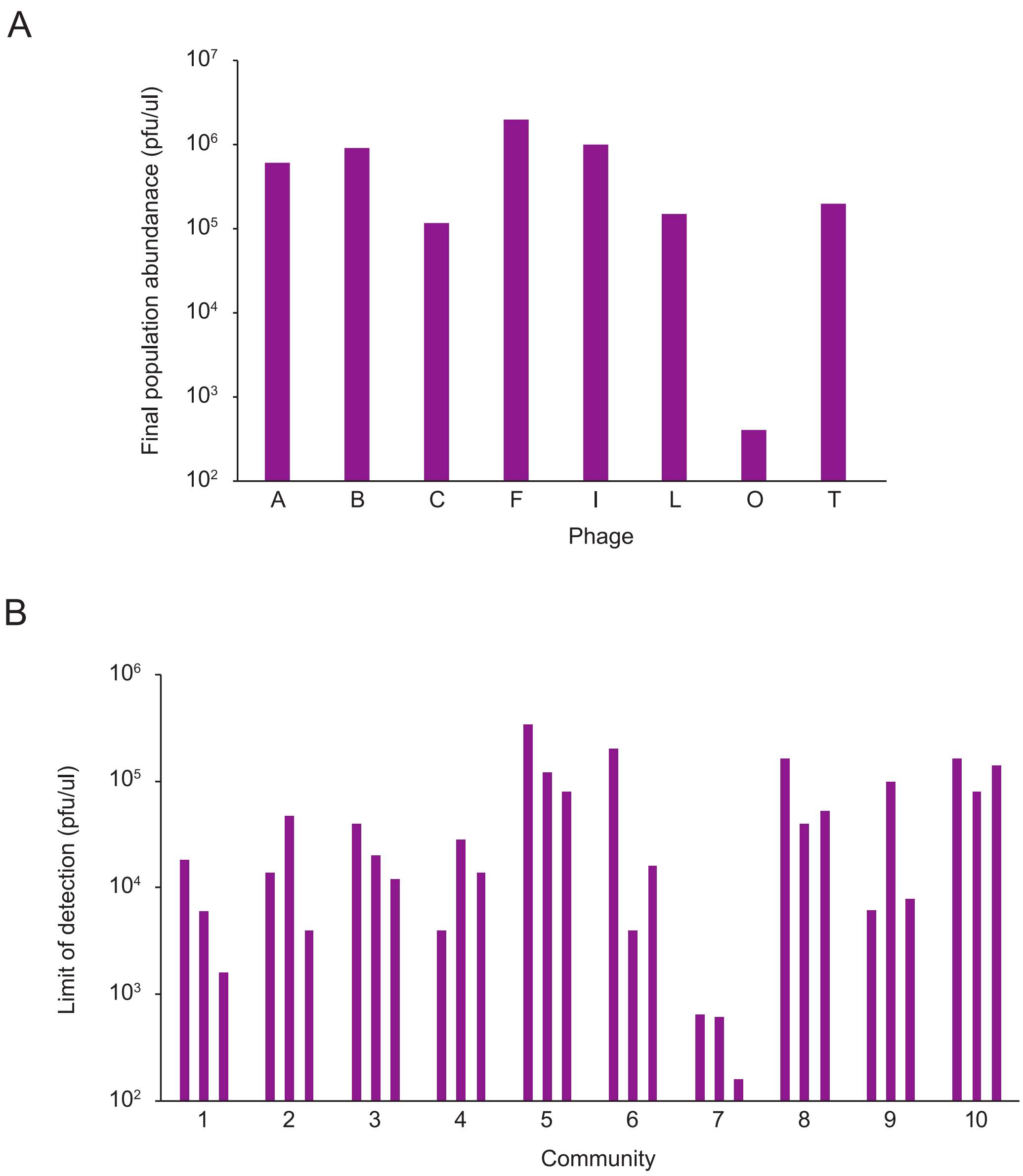

Fig. S6. Temperate species survived passaging in isolation.

(**A**) Temperate species were lost from all of our communities (fig. S5), however it was unclear whether their absence was due to their inherent inability to survive passaging or due to the presence of other phage species. We tested whether a subset of temperate species would be able to persist our passaging regime when passaged every 24 hours with filtering for 12 days. All species were detectable at the last passage, which suggested their absence in communities was due to competition with lytic species. (N = 1 biological replicate) (**B**) The limit of detection for an individual species in a community (pfu/ul) for the communities from Fig. 1E. This was calculated by multiplying the sequencing limit of detection (0.1%) with the total phage abundance for each community (Fig. 2A). The limit of detection for most communities (~10^4 pfu/ul) was lower than the abundance of most of the temperate species (10^5 or greater) during passaging (A). This

suggested that temperate species were below the limit of detection in communities due to the presence of competing phage species.

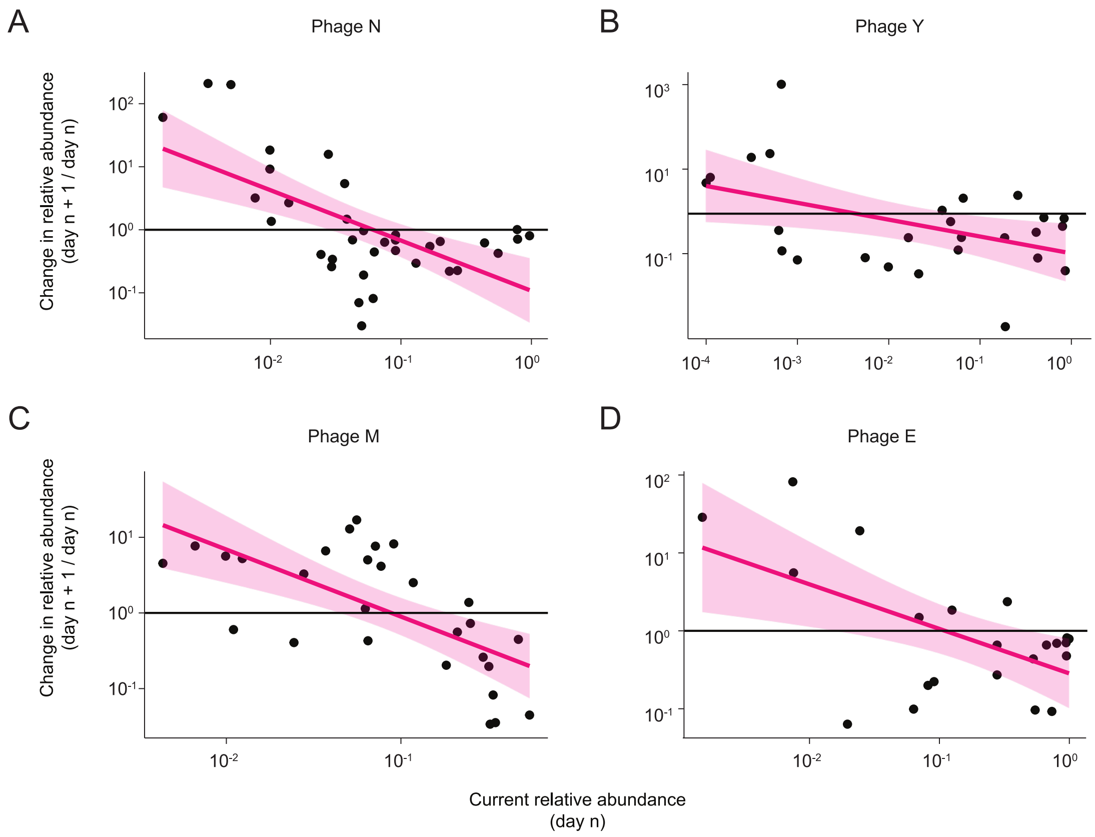

Fig. S7. Phage species in communities were under negative frequency dependent selection. To test for negative frequency dependent selection, we plotted the change in the relative abundance of one of the phage species in a community (listed in the subtitle) against that species' relative abundance using data from Fig. 2B for (**A-D**) communities 2, 3, 6, and 7. The y values were calculated by dividing the relative abundance of a species on the following passage (n + 1) by the abundance of that species at the current passage (n). For all four communities, this relationship was negative and y > 1 for low values of x. This indicates that both species in each community increased in frequency when rare (negative frequency dependence). The solid pink line was fitted to the data with a linear regression model and the shaded pink area indicates the 95% confidence interval. The regression line crosses y=1 (the thick black line) at x=0.080, 0.007, 0.110, 0.164 (from A-D) so a stable equilibrium containing both species exists at those relative abundances.

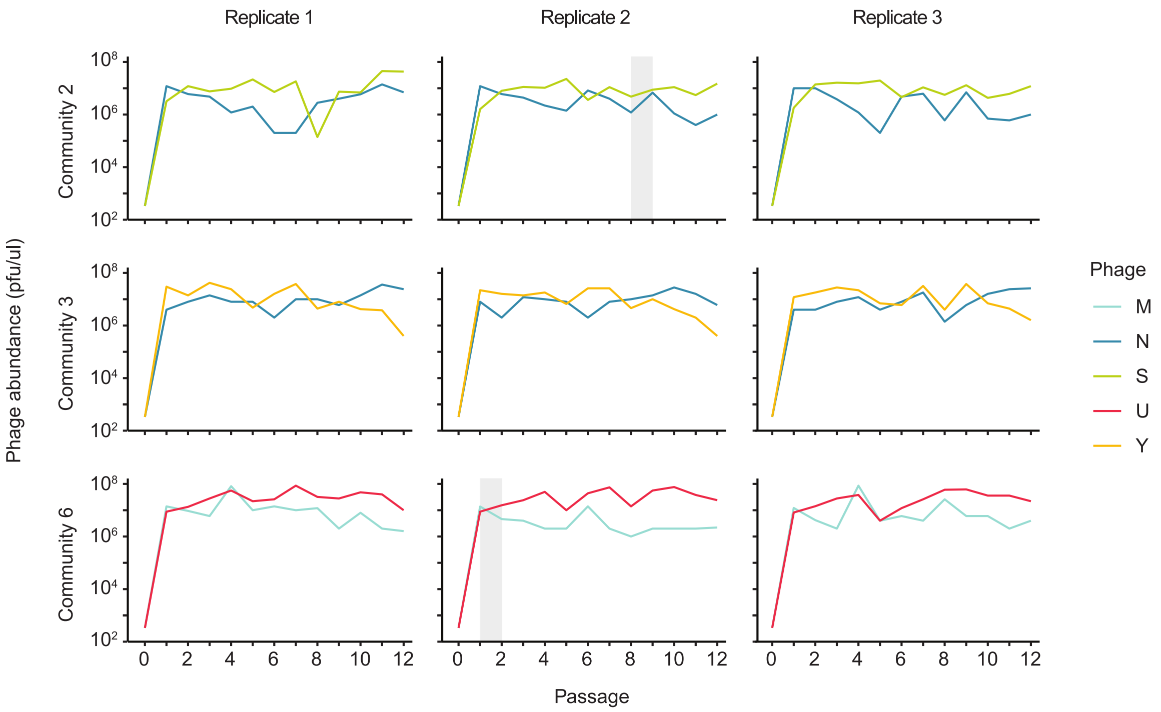

Fig. S8. Phage coexistence persisted in the absence of bacterial resistance evolution. We tested for the presence of phage resistant bacterial cells by plating the bacterial population during the passaging of three 2-phage communities from Fig. 2 (N = 3 biological replicates, left to right). We isolated colonies by plating the unfiltered communities at three time points (4 hours, 8 hours, and 24 hours) post-infection between passages 1-2, 4-5, 8-9, and 11-12. We also measured the phage abundance at the end of each passage through top agar plating. For most of the samples, no viable colonies were produced (plating 5 µl out of 300 µl per well, the limit of detection = 60 colonies per time point). However, we did detect bacterial growth in replicate 2 of community 2 between passages 8-9 and in replicate 2 of community 6 between passages 1-2 (shaded gray region). In both cases bacterial growth was detected starting at 4 hours post infection. We tested for phage resistance in these bacterial populations by culturing three colonies from each of these time points and top agar plating serial dilutions of phage stocks. In all cases the bacteria were resistant to both phage species in their resident community (phages N and S for community 2 or phages M and U for community 6) as evidenced by inhibited plaque formation compared to plating on the parental *E. coli* strain.

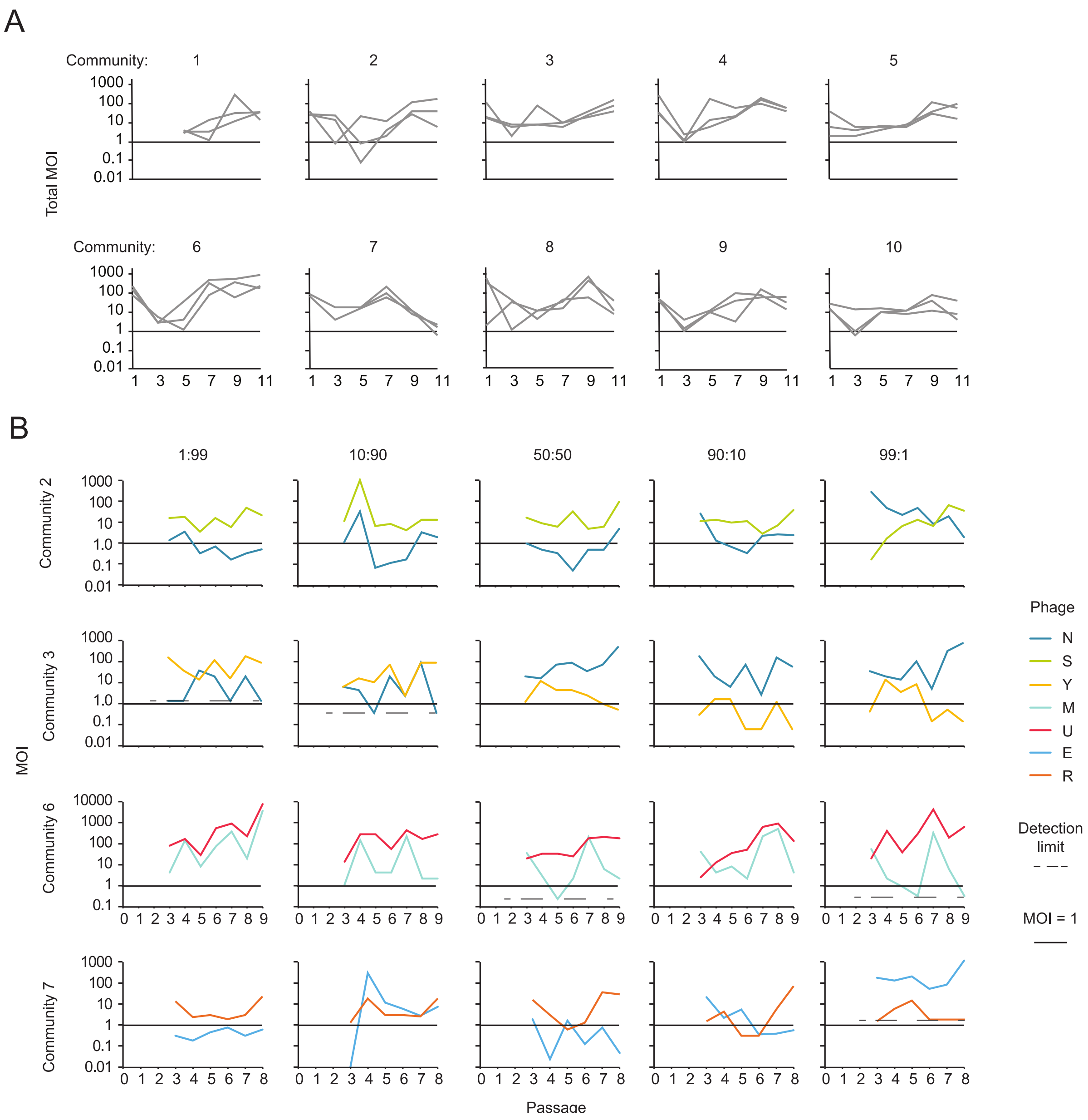

Fig. S9. Phages were more abundant than bacteria at the onset of each passage.
We determined whether the total phage population outnumbered bacteria (1.2 x 10^7 cfu into 300 µl) at the onset of each passage. When the ratio of pfu to cfu (ie. the MOI) is above y = 1 (the black line), then the phage population (pfu) exceeded the number of bacterial cells (cfu). Phage abundances were calculated through top agar plating for (**A**) the total phage population in three biological replicates of all 10 communities in Fig. 1E and (**B**) individual phage species from Fig. 2B.

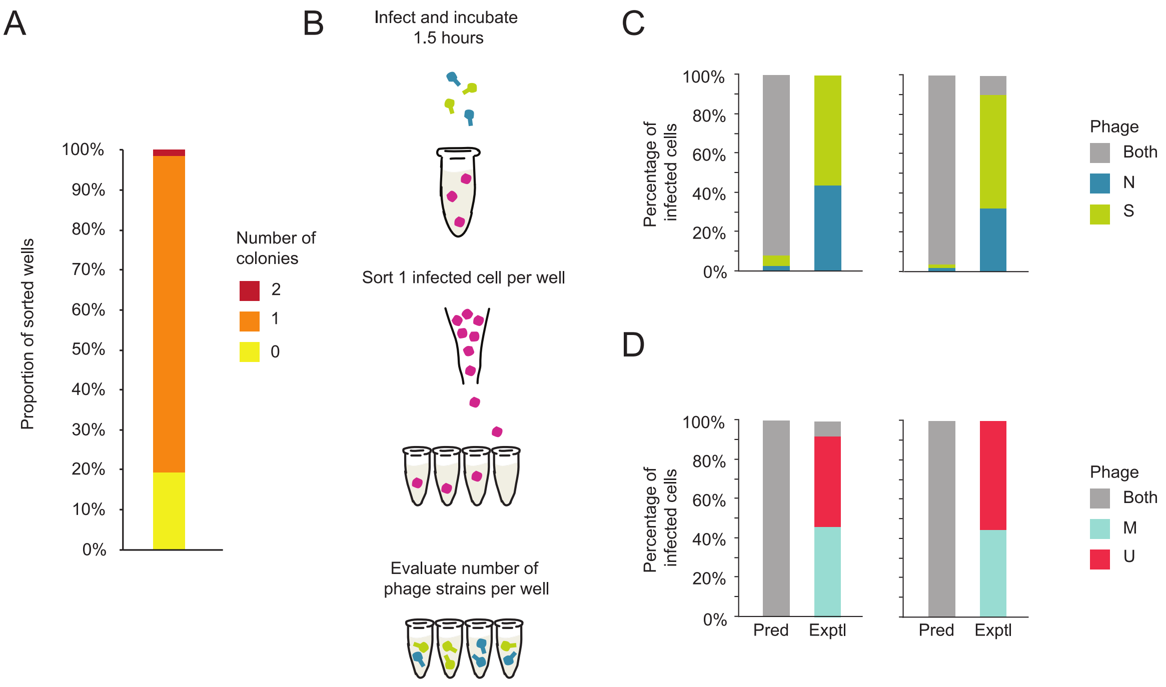

Fig. S10. Multiple phage species rarely reproduced within the same coinfected cells. The MOI of our communities was usually >1 (fig. S9) which suggested that individul cells might be productively coinfected by multiple phage species. We tested for productive coinfection using single-cell flow cytometry. (**A**) Validation that flow cytometry accurately sorts individual bacterial cells into each well. The proportion of wells that produced 0, 1, or 2 colonies after flow cytometry sorting. A single event was sorted into a well containing 20 µl of LB and then the entire contents of the well was plated onto LB agar and incubated to determine colony formation (N = 72 wells) (**B**) Schematic for determining the phage species produced from a single coinfected cell. Each co-infected cell was sorted just before lysis into a single well of a 96-well plate containing media. The phage species produced by each coinfected cell was determined through top agar plating. (**C**) Successful coinfection is rare, as seen in the low abundance of coinfected cells that produce one or two phage species. The predicted (pred) values were calculated using a Poisson distribution based on the MOI used during the experiments. The experimental results (exptl) were collected at 2 different MOIs for community 2 (left: MOI of 3.9, n = 16 cells; right: MOI of 4.8, n = 31 cells) and (**D**) community 7 (left: MOI of 25, n = 24 cells; right, MOI of 14, n = 9 cells).

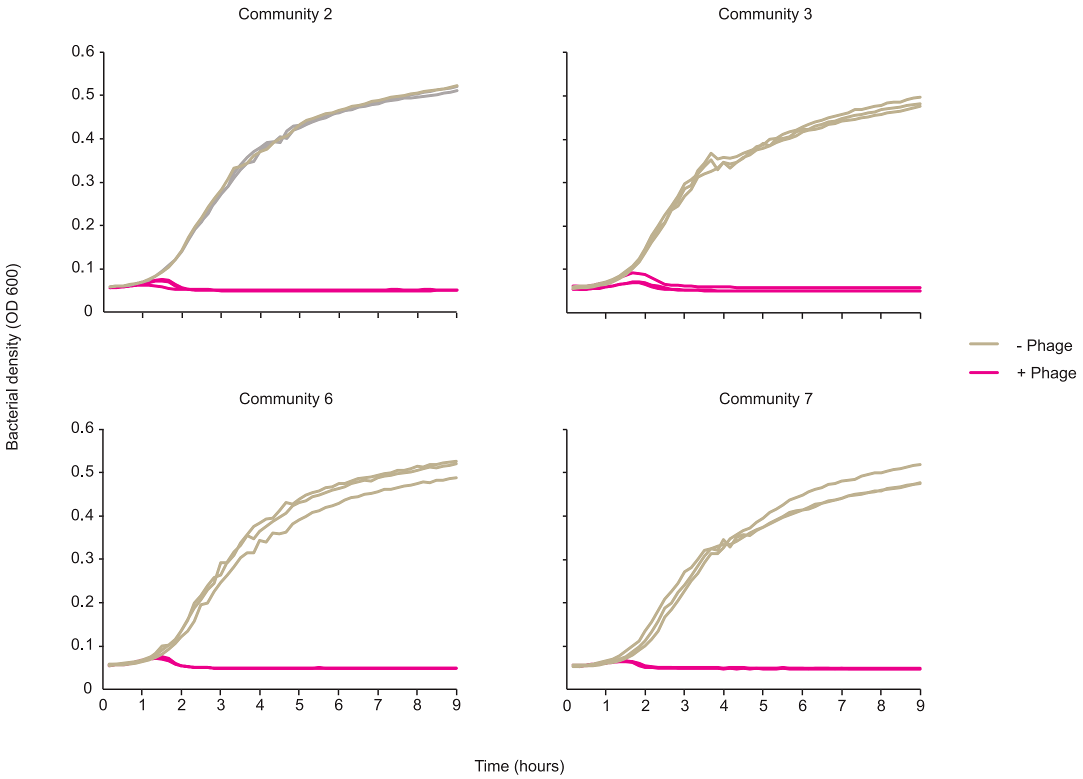

Fig. S11. Bacterial lysis occurred shortly after the onset of infection.
We determined the timing for bacterial lysis after infection by measure bacterial density through OD600 every 10 minutes during the first 9 hours after infection with or without phage communities (N = 3 biological replicates). Infections with a phage community showed an initial increase in OD600, followed by lysis around 1-2 hours after infection. No subsequent increase in OD was observed throughout the 24 hour incubation (even past the 9 hours shown here).

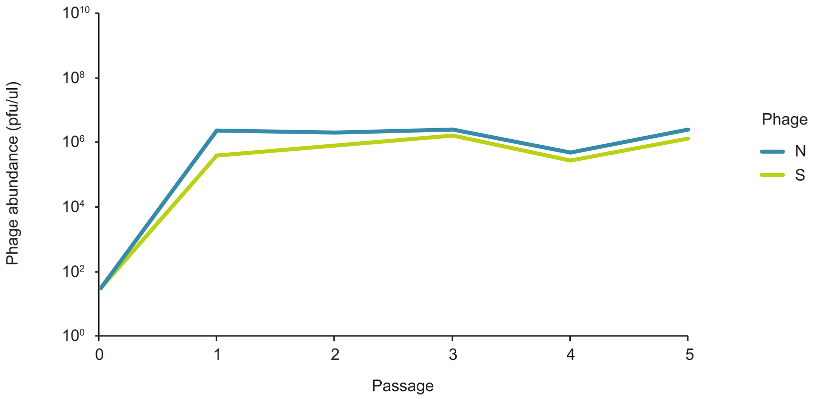

Fig. S12. Changing the bacterial culture inoculum increased the stability of community 2. Previous studies have found that cultures started from a frozen inoculum of *E. coli* have more consistent phage titers and more consistent *E.coli* phenotypes than cultures started with a single colony (*44*, *45*). We measured the abundances of phage N and S in community 2 through top agar plaquing when passaged on cultures started from a frozen stock. The abundance of phage N and S in community 2 showed less fluctuation from passage to passage than when infecting cultures started from a colony (Fig. 2A and B). Different working stocks of phage N and S were used to start the community in Fig. 2A and B, which may also have influenced the differences in fluctuations. The sample from passage 3 was used for the flow cytometry experiments in fig. S17 and Fig. 4D-F.

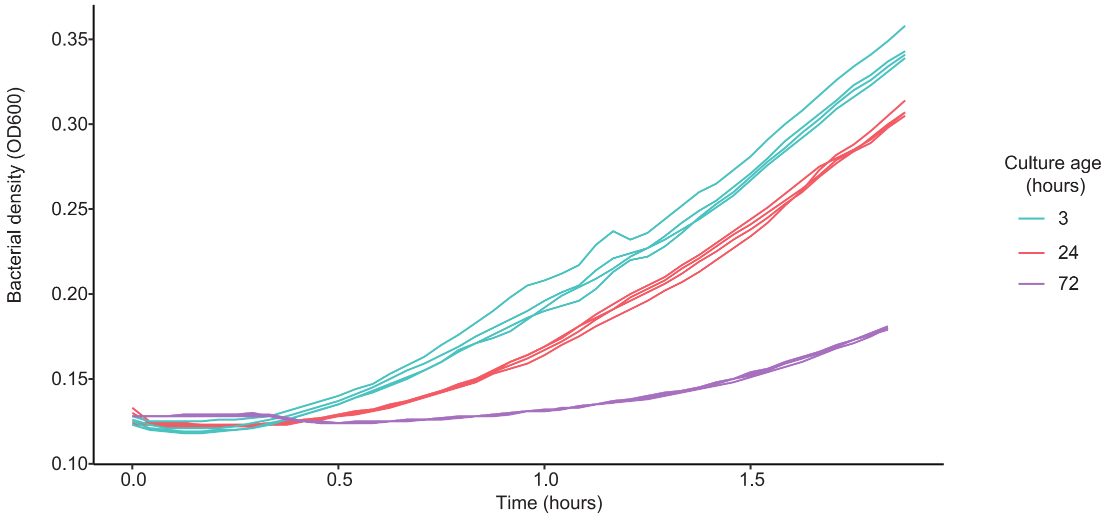

Fig S13. Older bacterial cultures regrew more slowly than younger cultures.
We measured bacterial density during regrowth of cultures in fresh LB. Each well contained 4 x 10^4 cfu/µl (final concentration) from cultures of different ages in 150 µl LB and was grown at 37°C. OD600 was measured every 2.5 minutes using an automated plate reader (N = 4 biological replicates).

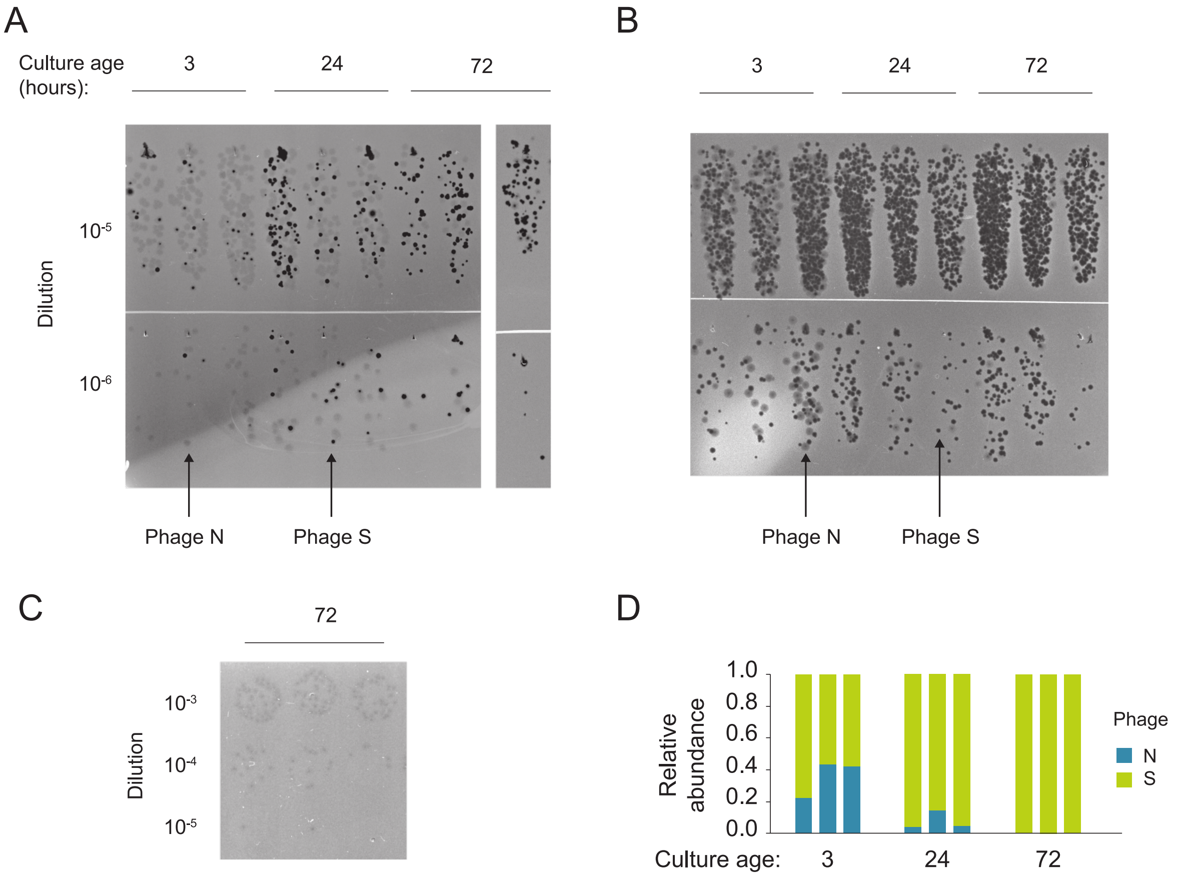

Fig. S14. Relative abundance of each species in community 2 depended on *E. coli* culture age during coinfection.

E. coli cultures of different ages (3, 24, or 72 hours) were infected with community 2. Plaque morphologies were used to determine the relative abundance of each phage species (N = 3 biological replicates, left to right). Each vertical section of the image is one biological replicate with 5ul plated per dilution. Arrows indicate the morphology of phage N or S. Before infections, the community was passaged for 1-2 days on a 24 hour culture to allow the species to reach equilibrium. Plaque morphologies for phage N and S varied slightly depending on the phage stock or due to plating conditions (i.e. the spread of bacteria across the plate or the batch of LB top agar) (**A**) Top agar plating for Fig. 4A (**B**) Replicate experiment of (A) using infections with a community generated from new stocks of phage N and S. (**C**) Phage N plaques were difficult to count in (B), so the dilution series was plated on a bacterial strain resistant to phage S. Each vertical section is 2ul plated from each dilution (**D**) Relative abundances of the phage N and S in the replicate experiment as quantified in (B). Phage N was not detectable at the -6 dilution**.** Phage N abundance decreased as culture age increased (for 3 hour, 24 hour, and 72 hour the mean = 0.37, 0.07, 0.00; 95% CI = 0.29-0.46, 0.0.03-0.13, 0.00-0.03). Shifting from the youngest culture (3 hours) to the oldest (72 hours) drastically altered the composition ($p<3.6e-11$, two-sample two-tailed z-test for equality of proportions) (N = 3). Phage N showed a relative preference for the younger culture, similar to Fig. 4A, however there was relatively more phage

S produced by all samples during this replicate experiment. This difference likely reflects the relative abundance of phage N and S in the passaged sample used to infect all cultures, which varied from passage to passage (Fig. 2).

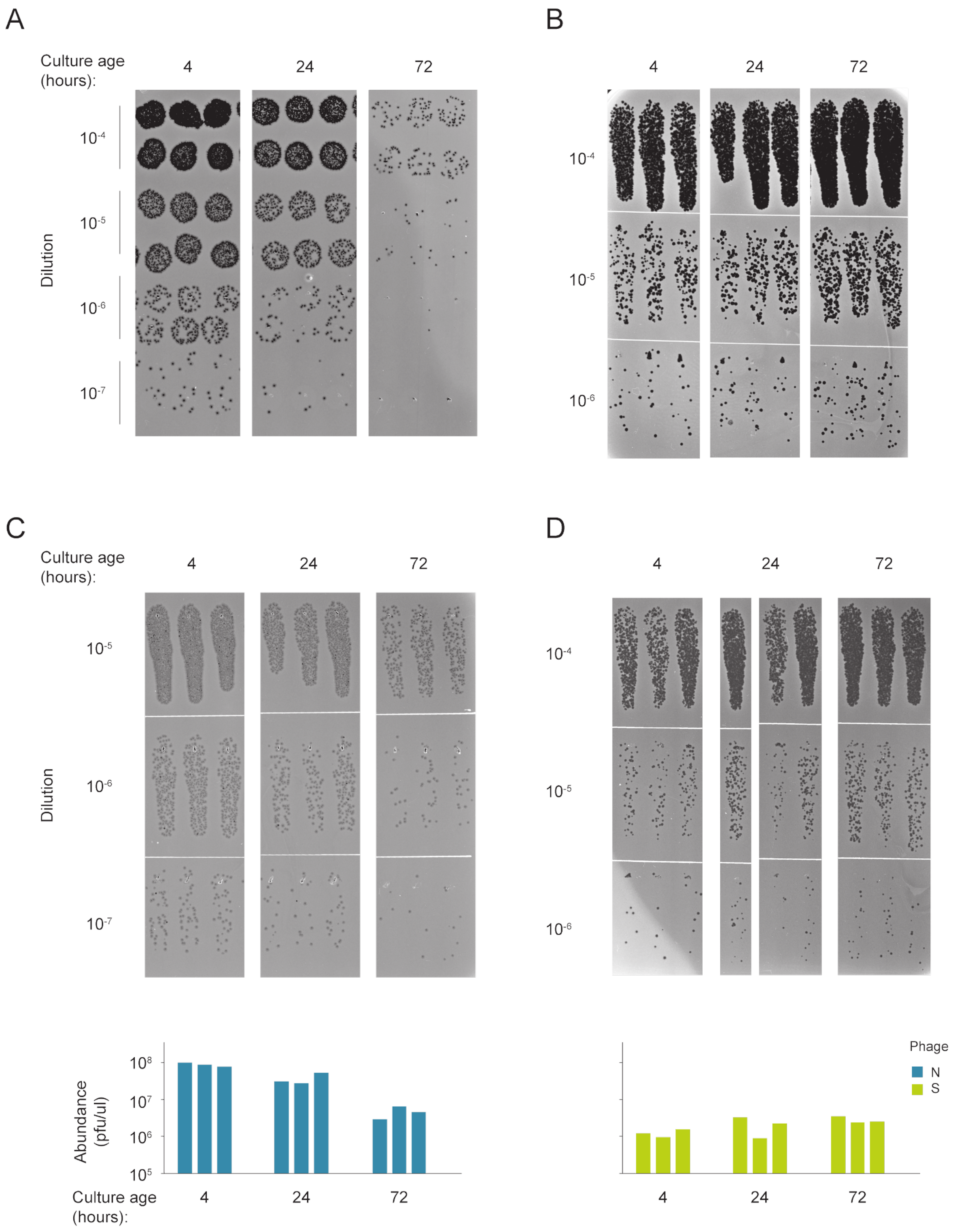
A
B
C
D
Culture age (hours):
4
24
72
Dilution
10^{-4}
10^{-5}
10^{-6}
10^{-7}
Abundance (pfu/ul)
10^{8}
10^{7}
10^{6}
10^{5}
Phage
N
S

Fig. S15. Abundance of phage N and S depended on *E. coli* culture age during solo infections.
E. coli cultures of different ages (4, 24, or 72 hours) were infected with either phage N or S and then abundances were measured through top agar plating (N = 3 biological replicates, left to right). Before infections, each species was passaged for 1-2 days on a 24 hour culture. Plaque morphologies for phage N and S varied slightly depending on the phage stock or due to plating conditions (i.e. the spread of bacteria across the plate or the batch of LB top agar) (**A**) Top agar plating for Fig. 4B top (phage N) Each vertical section of the image is one biological replicate with 5ul plated per dilution. (**B**) Top agar plating for Fig. 4B bottom (phage S). (**C**) Replicate experiment of (A) using new stocks of phage N. Consistent with our findings from Fig. 4B, grew to a higher abundance on the two younger cultures (3 hours and 24 hours) than it did on the oldest (72-hour) culture (adjusted $p = 0.010$ and $p < 0.001$ respectively, from one-tailed student's t-tests using the Benjamini-Hochberg correction for multiple comparisons). Phage N also grew to a higher abundance on the medium-aged culture (24 hour) than it did on the oldest culture (72 hour) (adjusted $p = 0.003$). (N = 3). (**D**) Replicate experiment of (B) using new stocks of phage S. Again consistent with Figure 4B, phage S grew to a higher abundance on the oldest culture compared to the youngest (adjusted $p = 0.010$), while achieving similar growth on the oldest and medium-age cultures, and on the medium-age and youngest cultures (adjusted $p = 0.195$ and adjusted $p = 0.195$ respectively). (N = 3).

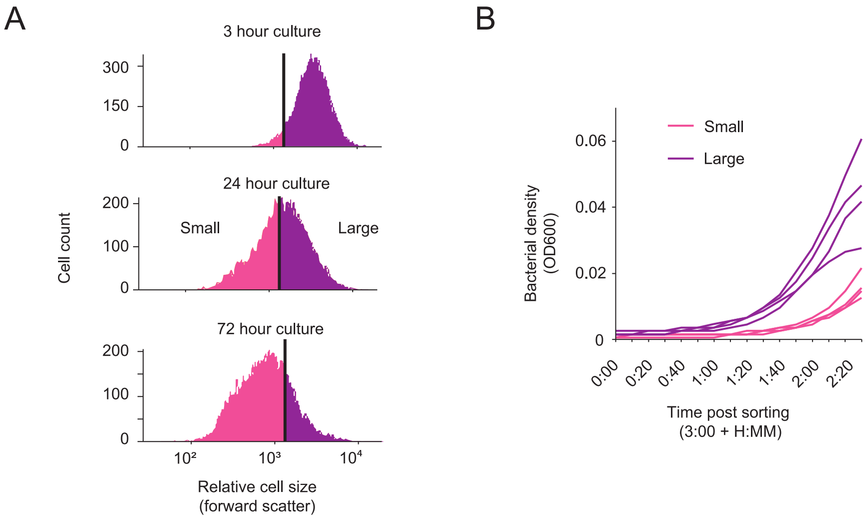

Fig. S16. Cell size was an accurate marker for growth physiology.
(**A**) Validation that older cultures had a greater fraction of smaller sized *E. coli* cells (*28*, *31*). Flow cytometry forward scatter analyses on 3 hour, 24 hour, and 72 hour cultures (top to bottom). The thick black line divides ~50% of cells from the 24 hour culture. (**B**) Small cells grew slower than large cells from the same 24 hour culture. Small or large cells (100 cells per biological replicate) were sorted into 4 wells of a 96-well plate containing LB media by gating events with the highest 30% (large cells) or lowest 30% (small cells) forward scatter. OD600 was measured every 10 minutes using an automated plate reader.

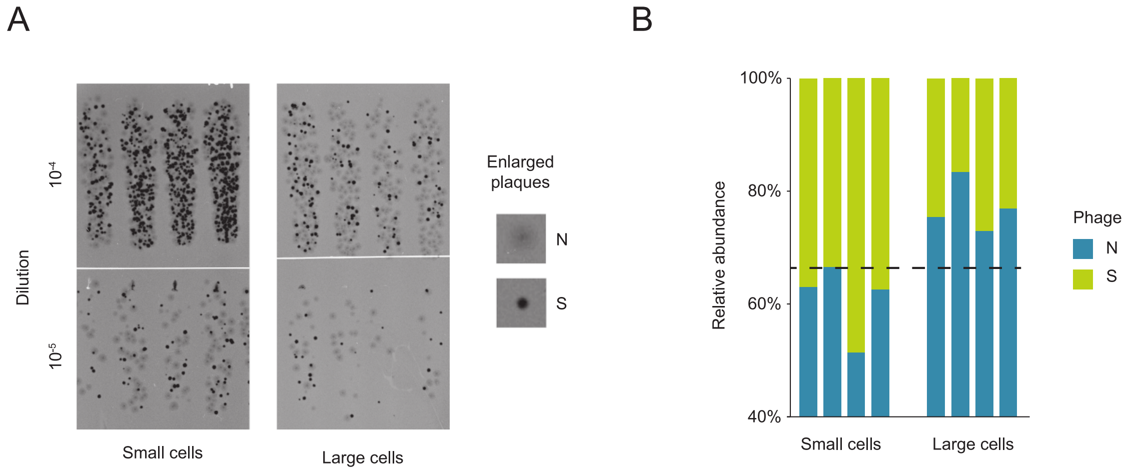

Fig. S17. Small and large bacterial subpopulations preferrentially produced different phage species.
Replicate experiment of Fig. 4D and E from an independent flow cytometry run (N = 4 biological replicates). Bacterial populations were infected using the same phage sample as Fig. 4 (community 2 at passage 3 of fig. S12). (**A**) Top agar plating of phage species produced by cells of different sizes. (**B**) Relative abundance of each phage species, with the dotted line indicating the starting proportion of phage N.

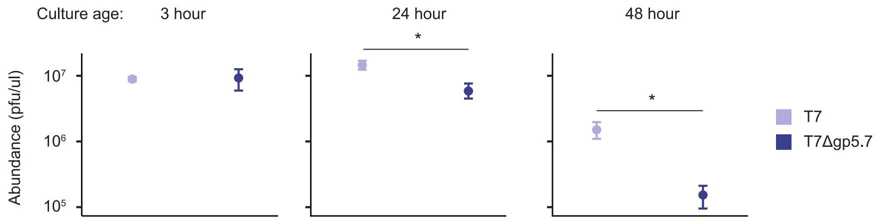

Fig. S18. The abundance of T7 on different cultures was regulated by phage-encoded *gp5.7*. To validate that T7Δ*gp5.7* had a fitness disadvantage relative to wild type T7 on older cultures (*29*), we infected cultures of different ages with T7 or T7Δ*gp5.7* (N = 3 biological replicates). Infections were done at an MOI of 5.0 and phage abundances were then measured through plaque morphology after 24 hours incubation. T7Δ*gp5.7* was significantly less abundant than the wild-type T7 on the older cultures (24 hour and 48 hour), but had a similar abundance on the 3 hour culture. Light blue dot and error bars represent the mean ± SE for wild type T7, while dark blue dot and error bars represent T7Δ*gp5.7*. Starred comparisons had *P* values at 0.037 and 0.035 (two-tailed t-test, N=4)

A

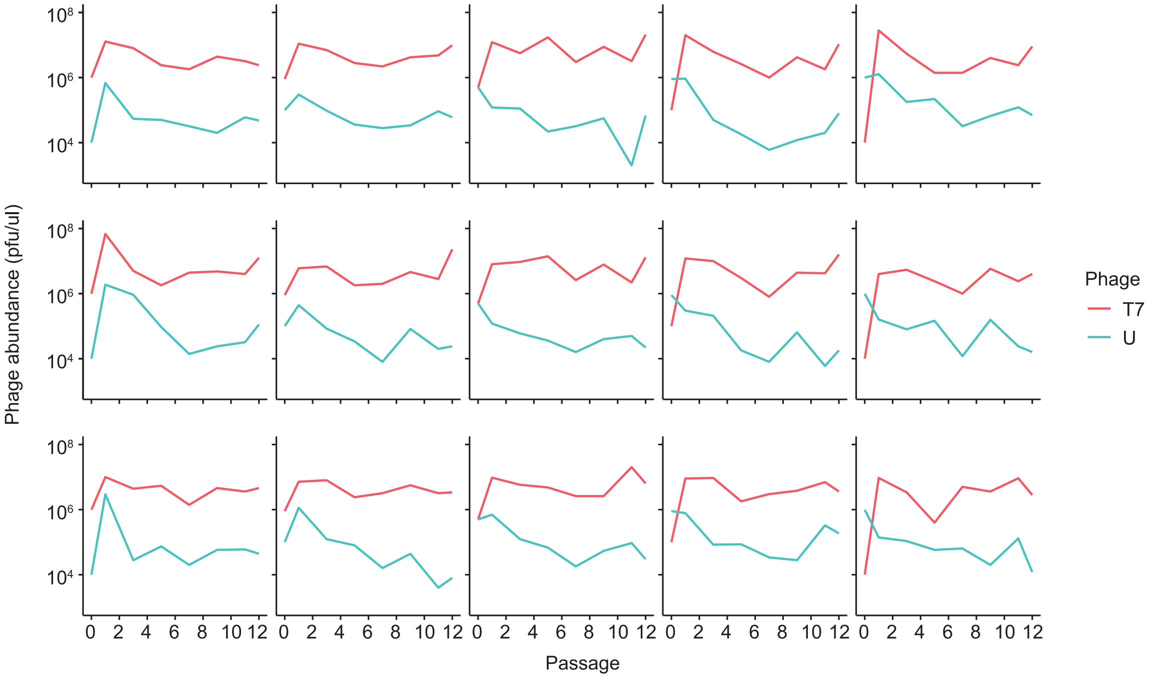
1:99
10:90
50:50
90:10
99:1
Phage abundance (pfu/ul)
Passage
Phage
T7
U

B

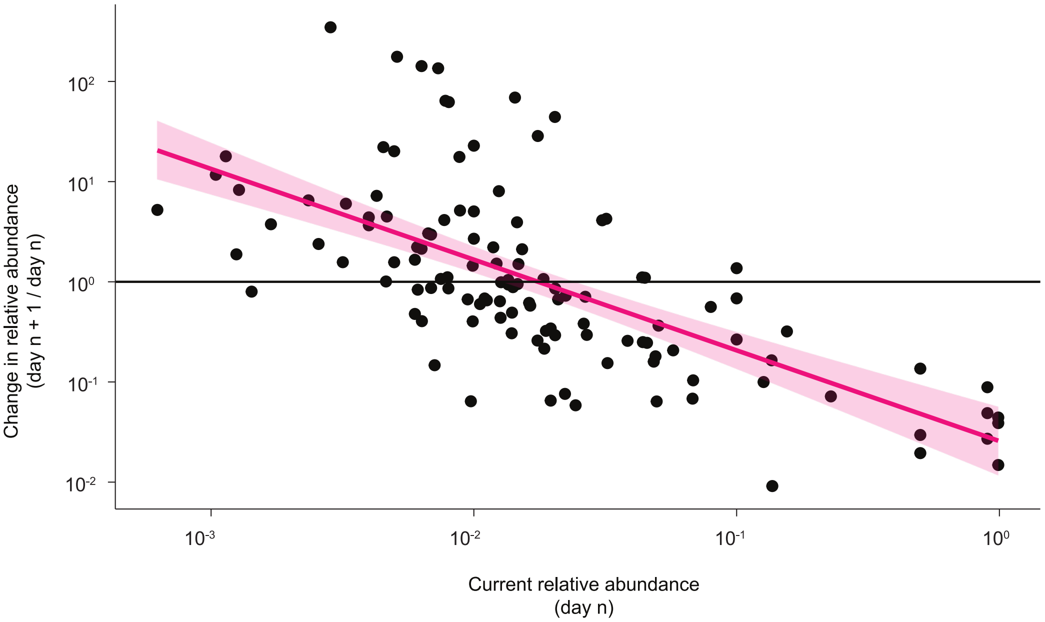
Change in relative abundance
(day n + 1 / day n)
Current relative abundance
(day n)

Fig. S19. Phage T7 was stable in community with phage U.

(**A**) The abundances of phage U and T7 during passaging of communities with different starting relative abundances of each phage, as measured by top agar. Communities were stable throughout the passaging, even when each species was started from a rare proportion of the population. (N = 3 biological replicates, shown top to bottom). The initial relative abundances of each phage species (listed from left to right, phage U:T7) varied from ~ 1% to ~ 99% of the total phage population. Phages were titered on passage 0 and then every other day starting at passage 1 until passage 11 and 12. To quantify phage U, samples were also titered on an *E. coli* strain resistant to T7 (Methods). (**B**) Using the data from (A) we tested for negative frequency dependent selection which is a hallmark of a stable community. The change in relative abundance of phage U (in community with T7) (y-axis) is plotted against phage U's relative abundance (x-axis). The y-axis is calculated by dividing the relative abundance of phage U at the end of the following passage (n + 1) by the abundance of that phage U at the end of the current passage (n). The relationship between both was negative, and y > 1 for low values of x. This indicated that both phages U and T7 increased in frequency when rare (i.e. negative frequency dependent selection). The solid pink line was fitted to the data with a linear regression model and the shaded pink area indicates the 95% confidence interval. Since the regression line has a negative slope and it crosses y=1 (the thick black line) at x=0.017, a stable equilibrium containing both phage species existed at that relative abundance.

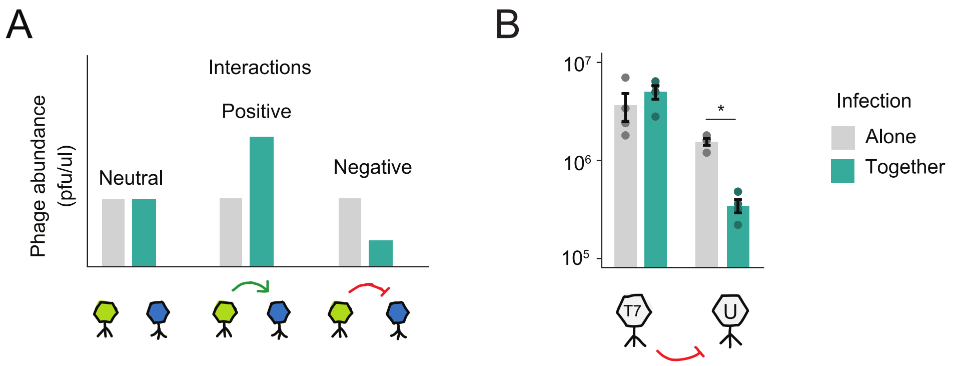

Fig. S20. Interactions between T7 and phage U were negative.
(**A**) Example results that show how ecological interactions are determined through changes in phage abundance. The green phage can either have no impact, increase, or decrease the blue phage's abundance during coinfection, relative to when the blue phage is infecting alone. These interactions would be neutral, positive, or negative, respectively. All samples were passaged for 3 days before measuring the phage abundances through top agar plating. (**B**) Phage abundances after an infection together versus alone. Gray dots represent the abundance of each phage in each of 4 independent biological replicates. Bars represent the mean of the N=4 biological replicates, and the error bars represent one standard deviation. Starred sample had a *P* value <0.001 (two-tailed t-test, N=4)

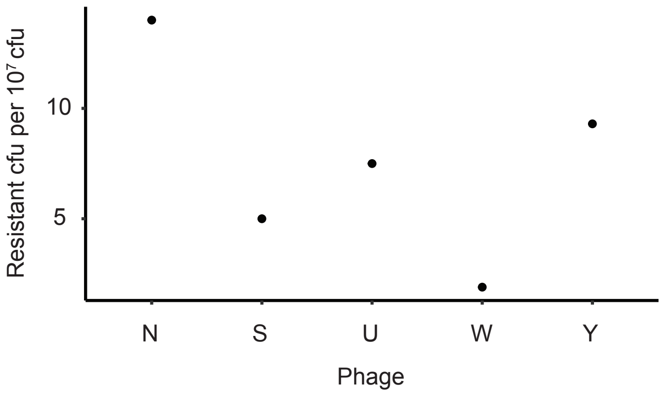

Fig. S21. Phage-resistance rates in the bacterial population.
We did not see evidence of phage-resistant bacterial mutants during community infection (fig. S11), however we next determined their abundance at the onset of infection. We calculated the rates of phage resistance against a subset of individual phage species by quantifying the number of surviving colonies after infecting a top agar lawn with each species. Across the different phages, the average rate of phage resistance was ~1 in 10^6 cfu. At the onset of passaging, ~10 resistant bacteria would be present per well (1.2 x 10^7 total cfu in 300ul) and infected by phage at ~ 6 logs higher abundance (Fig. 2), so these resistant mutants were unlikely to contribute to the coexistence of multiple phage species in our communities.

Data table S1. Phage collection characteristics. Lytic and temperate bioinformatics classifications refer to (A) BACPHLIP (*53*) and (B) PhaTYP (*52*).

Data table S2. Sequence of primers and plasmid insertions used in this study.
